# Supplementary figures and images for: The transcriptional corepressor CTBP-1 acts with the SOX family transcription factor EGL-13 to maintain AIA interneuron cell identity in Caenorhabditis elegans
Source: eLife. 2022 Feb 4;11:e74557. doi: 10.7554/eLife.74557 (PMC8816384; doi:10.7554/eLife.74557)

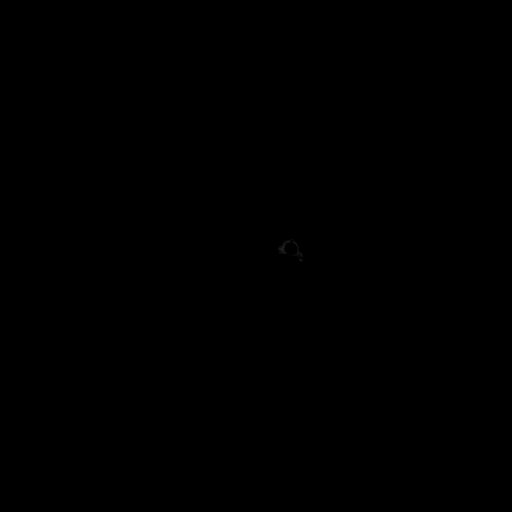

Supplement: Figure 5—source data 1. [file elife-74557-fig5-data1.zip › Saul_et_al_2022_Figure 5/Fig 5/Fig 5C_images/nIs348;ctbp-1(n4784);otIs123/L4/Snap-371.tiff_files/Snap-371_h0b0t0z0c0x0-512y0-512.tiff]

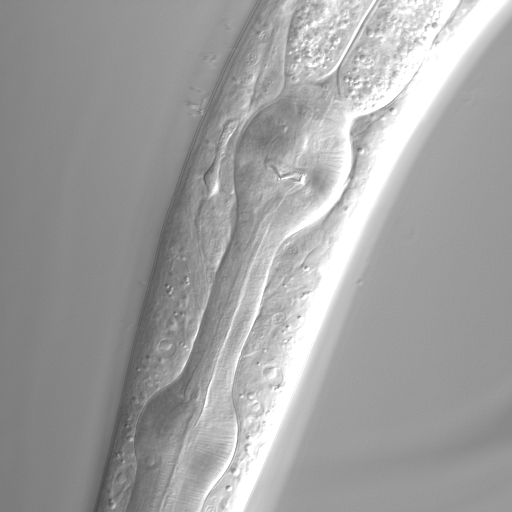

Supplement: Figure 5—source data 1. [file elife-74557-fig5-data1.zip › Saul_et_al_2022_Figure 5/Fig 5/Fig 5C_images/nIs348;ctbp-1(n4784);otIs123/L4/Snap-371.tiff_files/Snap-371_h0b0t0z0c1x0-512y0-512.tiff]

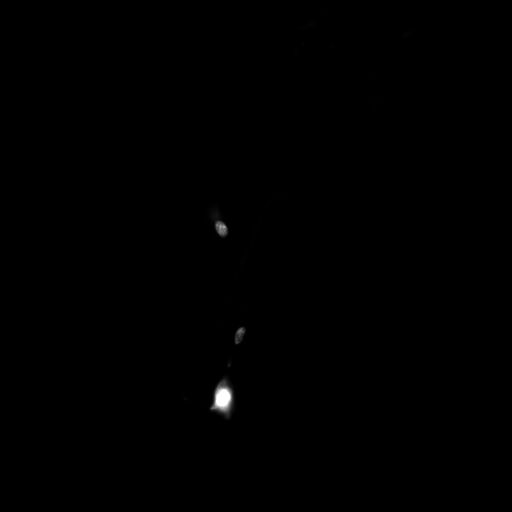

Supplement: Figure 5—source data 1. [file elife-74557-fig5-data1.zip › Saul_et_al_2022_Figure 5/Fig 5/Fig 5C_images/nIs348;ctbp-1(n4784);otIs123/L4/Snap-371.tiff_files/Snap-371_h0b0t0z0c2x0-512y0-512.tiff]

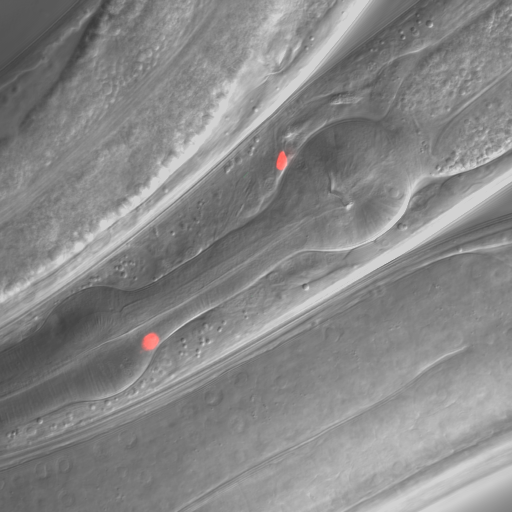

Supplement: Figure 5—source data 1. [file elife-74557-fig5-data1.zip › Saul_et_al_2022_Figure 5/Fig 5/Fig 5C_images/nIs348;ctbp-1(n4784);otIs123/L4/Snap-376.tiff]

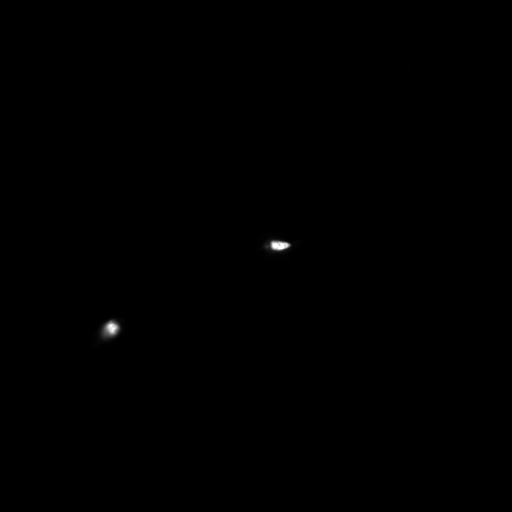

Supplement: Figure 5—source data 1. [file elife-74557-fig5-data1.zip › Saul_et_al_2022_Figure 5/Fig 5/Fig 5C_images/nIs348;ctbp-1(n4784);otIs123/L4/Snap-379.tiff_files/Snap-379_h0b0t0z0c2x0-512y0-512.tiff]

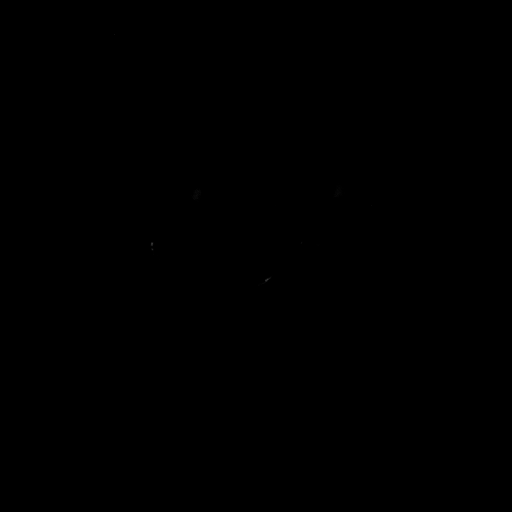

Supplement: Figure 5—source data 1. [file elife-74557-fig5-data1.zip › Saul_et_al_2022_Figure 5/Fig 5/Fig 5C_images/nIs348;ctbp-1(n4784);otIs123/L4/Snap-379.tiff_files/Snap-379_h0b0t0z0c0x0-512y0-512.tiff]

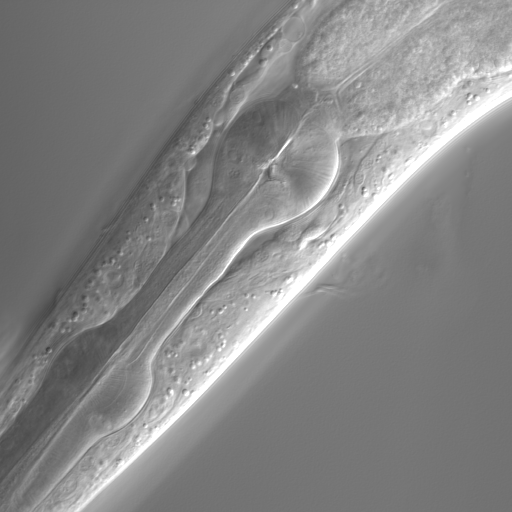

Supplement: Figure 5—source data 1. [file elife-74557-fig5-data1.zip › Saul_et_al_2022_Figure 5/Fig 5/Fig 5C_images/nIs348;ctbp-1(n4784);otIs123/L4/Snap-379.tiff_files/Snap-379_h0b0t0z0c1x0-512y0-512.tiff]

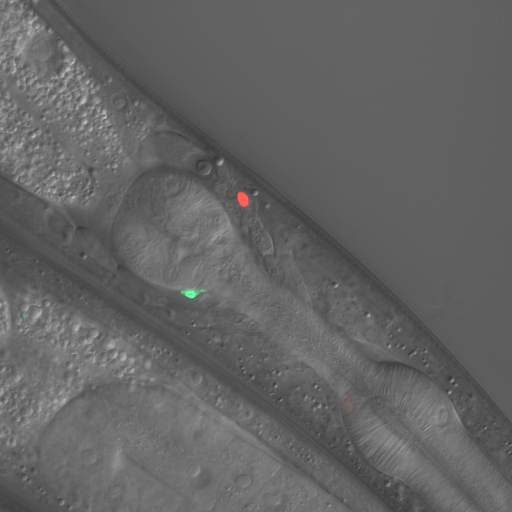

Supplement: Figure 5—source data 1. [file elife-74557-fig5-data1.zip › Saul_et_al_2022_Figure 5/Fig 5/Fig 5C_images/nIs348;ctbp-1(n4784);otIs123/L4/Snap-377.tiff]

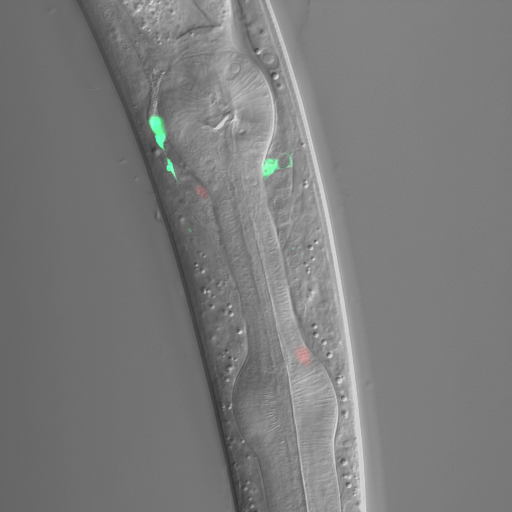

Supplement: Figure 5—source data 1. [file elife-74557-fig5-data1.zip › Saul_et_al_2022_Figure 5/Fig 5/Fig 5C_images/nIs348;ctbp-1(n4784);otIs123/L4/Snap-370.tiff]

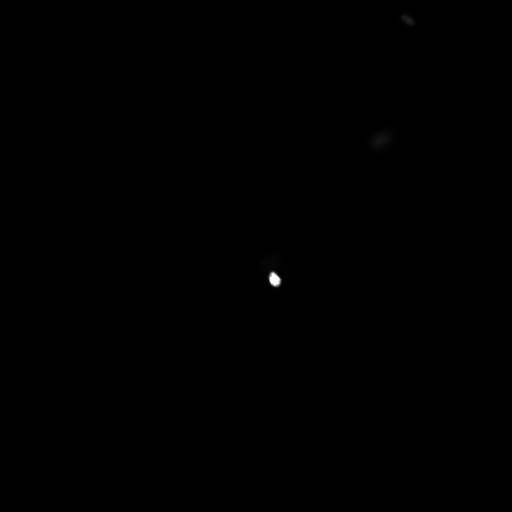

Supplement: Figure 5—source data 1. [file elife-74557-fig5-data1.zip › Saul_et_al_2022_Figure 5/Fig 5/Fig 5C_images/nIs348;ctbp-1(n4784);otIs123/L4/Snap-373.tiff_files/Snap-373_h0b0t0z0c2x0-512y0-512.tiff]

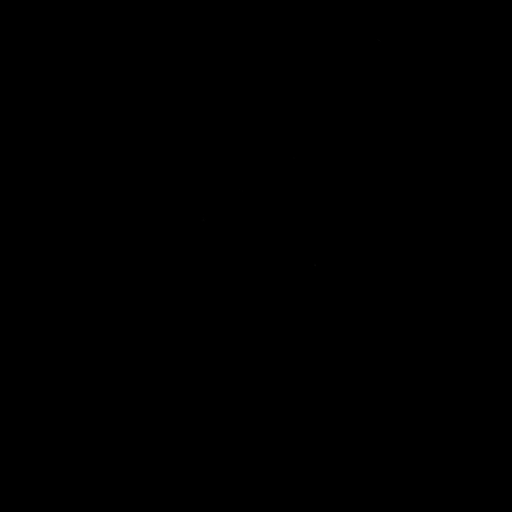

Supplement: Figure 5—source data 1. [file elife-74557-fig5-data1.zip › Saul_et_al_2022_Figure 5/Fig 5/Fig 5C_images/nIs348;ctbp-1(n4784);otIs123/L4/Snap-373.tiff_files/Snap-373_h0b0t0z0c0x0-512y0-512.tiff]

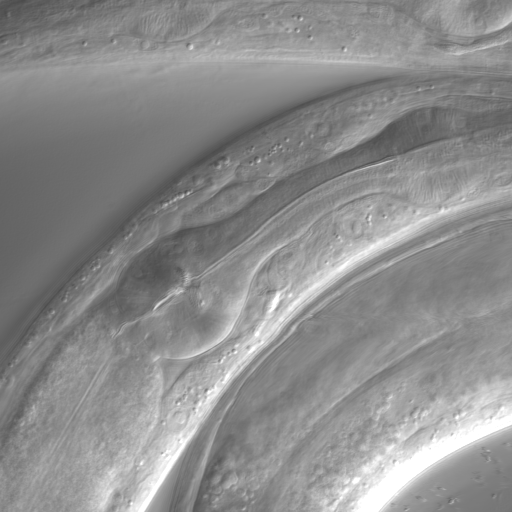

Supplement: Figure 5—source data 1. [file elife-74557-fig5-data1.zip › Saul_et_al_2022_Figure 5/Fig 5/Fig 5C_images/nIs348;ctbp-1(n4784);otIs123/L4/Snap-373.tiff_files/Snap-373_h0b0t0z0c1x0-512y0-512.tiff]

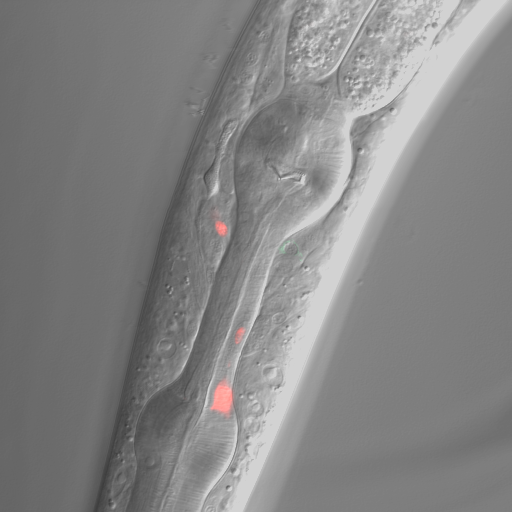

Supplement: Figure 5—source data 1. [file elife-74557-fig5-data1.zip › Saul_et_al_2022_Figure 5/Fig 5/Fig 5C_images/nIs348;ctbp-1(n4784);otIs123/L4/Snap-371.tiff]

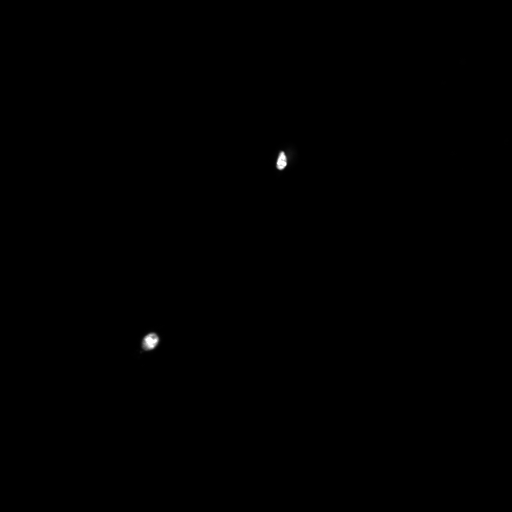

Supplement: Figure 5—source data 1. [file elife-74557-fig5-data1.zip › Saul_et_al_2022_Figure 5/Fig 5/Fig 5C_images/nIs348;ctbp-1(n4784);otIs123/L4/Snap-376.tiff_files/Snap-376_h0b0t0z0c2x0-512y0-512.tiff]

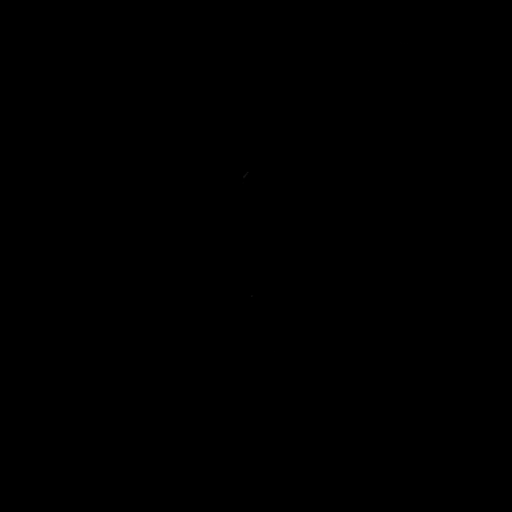

Supplement: Figure 5—source data 1. [file elife-74557-fig5-data1.zip › Saul_et_al_2022_Figure 5/Fig 5/Fig 5C_images/nIs348;ctbp-1(n4784);otIs123/L4/Snap-376.tiff_files/Snap-376_h0b0t0z0c0x0-512y0-512.tiff]

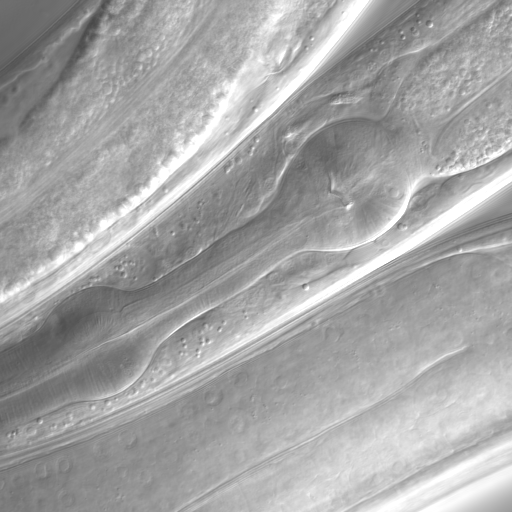

Supplement: Figure 5—source data 1. [file elife-74557-fig5-data1.zip › Saul_et_al_2022_Figure 5/Fig 5/Fig 5C_images/nIs348;ctbp-1(n4784);otIs123/L4/Snap-376.tiff_files/Snap-376_h0b0t0z0c1x0-512y0-512.tiff]

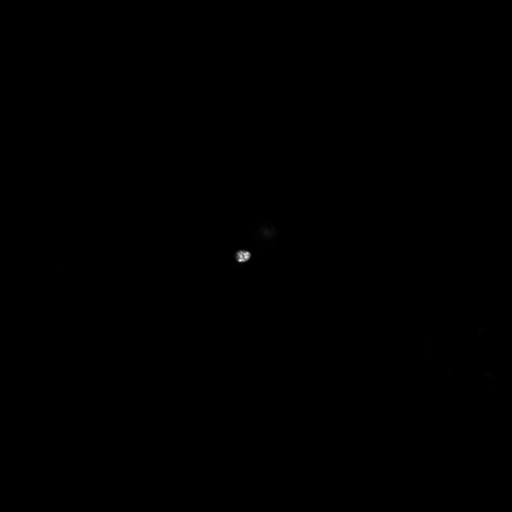

Supplement: Figure 5—source data 1. [file elife-74557-fig5-data1.zip › Saul_et_al_2022_Figure 5/Fig 5/Fig 5C_images/nIs348;ctbp-1(n4784);otIs123/L4/Snap-378.tiff_files/Snap-378_h0b0t0z0c2x0-512y0-512.tiff]

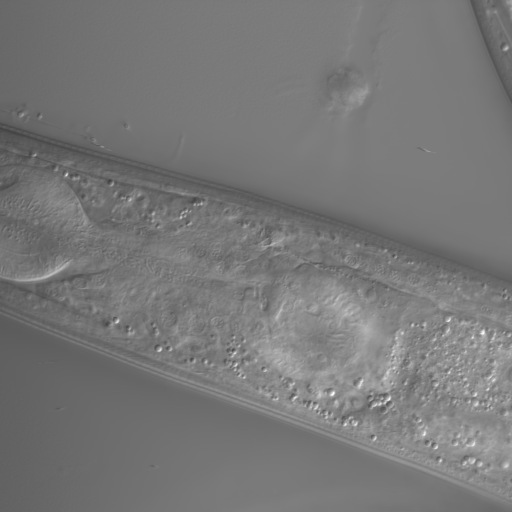

Supplement: Figure 5—source data 1. [file elife-74557-fig5-data1.zip › Saul_et_al_2022_Figure 5/Fig 5/Fig 5C_images/nIs348;ctbp-1(n4784);otIs123/L4/Snap-378.tiff_files/Snap-378_h0b0t0z0c1x0-512y0-512.tiff]

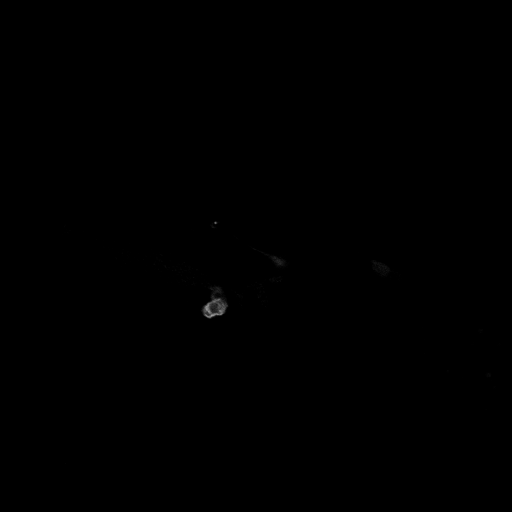

Supplement: Figure 5—source data 1. [file elife-74557-fig5-data1.zip › Saul_et_al_2022_Figure 5/Fig 5/Fig 5C_images/nIs348;ctbp-1(n4784);otIs123/L4/Snap-378.tiff_files/Snap-378_h0b0t0z0c0x0-512y0-512.tiff]

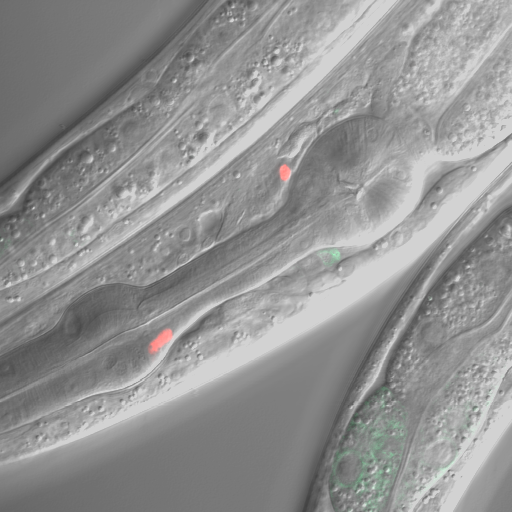

Supplement: Figure 5—source data 1. [file elife-74557-fig5-data1.zip › Saul_et_al_2022_Figure 5/Fig 5/Fig 5C_images/nIs348;ctbp-1(n4784);otIs123/L4/Snap-372.tiff]

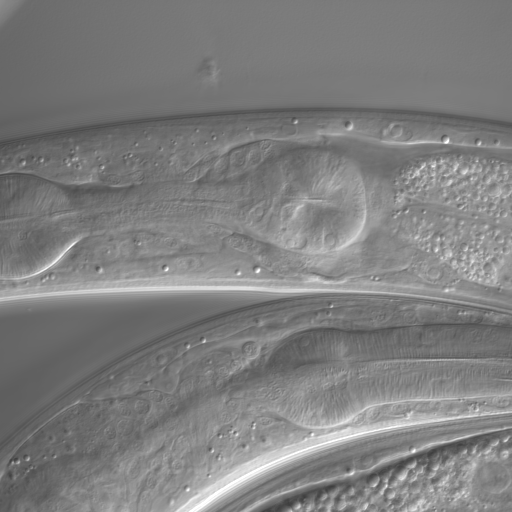

Supplement: Figure 5—source data 1. [file elife-74557-fig5-data1.zip › Saul_et_al_2022_Figure 5/Fig 5/Fig 5C_images/nIs348;ctbp-1(n4784);otIs123/L4/Snap-375.tiff_files/Snap-375_h0b0t0z0c1x0-512y0-512.tiff]

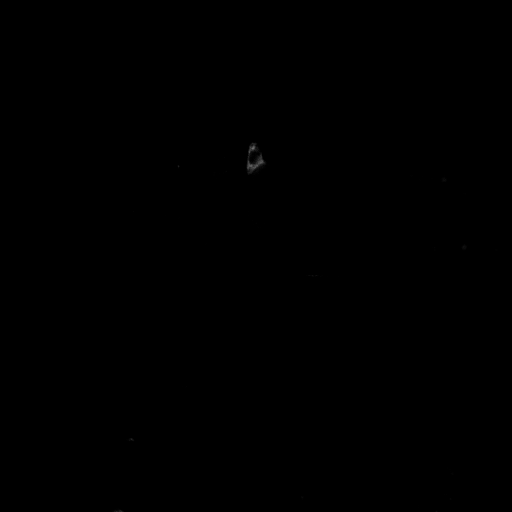

Supplement: Figure 5—source data 1. [file elife-74557-fig5-data1.zip › Saul_et_al_2022_Figure 5/Fig 5/Fig 5C_images/nIs348;ctbp-1(n4784);otIs123/L4/Snap-375.tiff_files/Snap-375_h0b0t0z0c0x0-512y0-512.tiff]

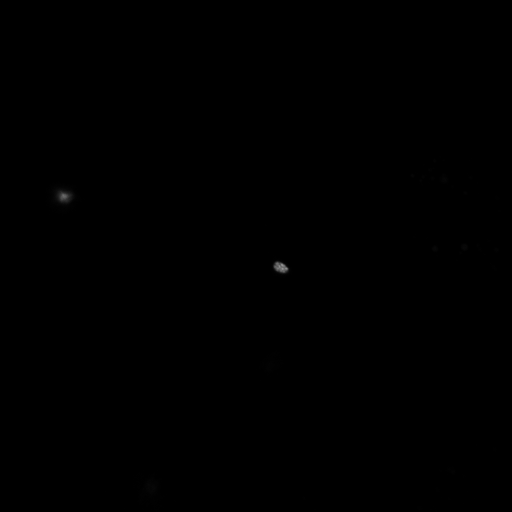

Supplement: Figure 5—source data 1. [file elife-74557-fig5-data1.zip › Saul_et_al_2022_Figure 5/Fig 5/Fig 5C_images/nIs348;ctbp-1(n4784);otIs123/L4/Snap-375.tiff_files/Snap-375_h0b0t0z0c2x0-512y0-512.tiff]

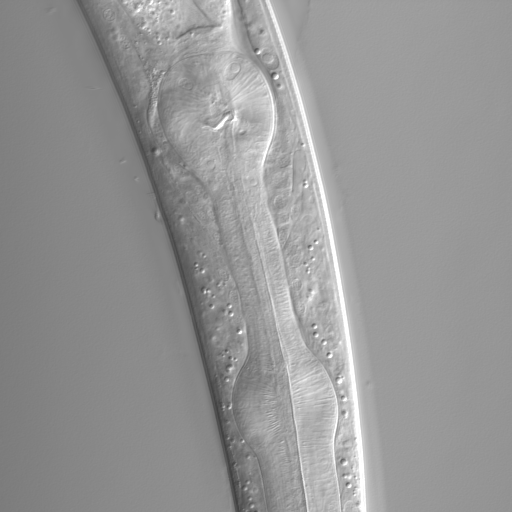

Supplement: Figure 5—source data 1. [file elife-74557-fig5-data1.zip › Saul_et_al_2022_Figure 5/Fig 5/Fig 5C_images/nIs348;ctbp-1(n4784);otIs123/L4/Snap-370.tiff_files/Snap-370_h0b0t0z0c1x0-512y0-512.tiff]

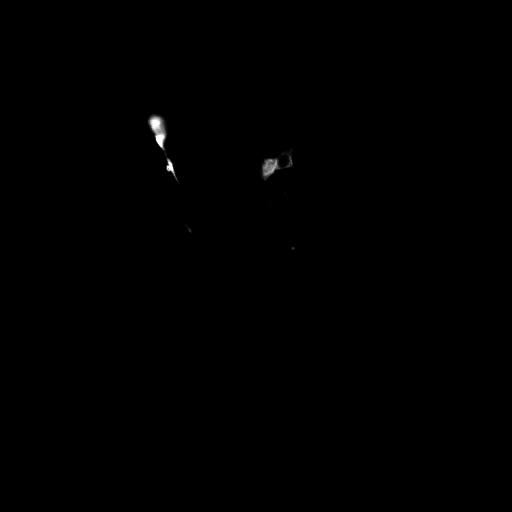

Supplement: Figure 5—source data 1. [file elife-74557-fig5-data1.zip › Saul_et_al_2022_Figure 5/Fig 5/Fig 5C_images/nIs348;ctbp-1(n4784);otIs123/L4/Snap-370.tiff_files/Snap-370_h0b0t0z0c0x0-512y0-512.tiff]

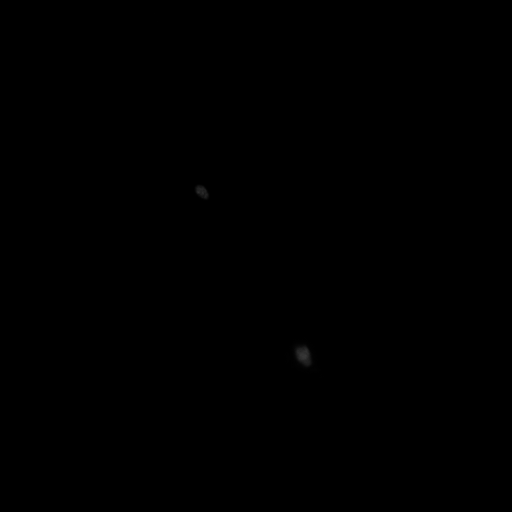

Supplement: Figure 5—source data 1. [file elife-74557-fig5-data1.zip › Saul_et_al_2022_Figure 5/Fig 5/Fig 5C_images/nIs348;ctbp-1(n4784);otIs123/L4/Snap-370.tiff_files/Snap-370_h0b0t0z0c2x0-512y0-512.tiff]

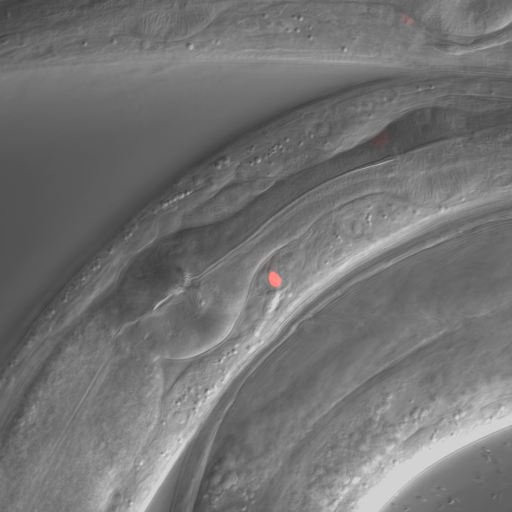

Supplement: Figure 5—source data 1. [file elife-74557-fig5-data1.zip › Saul_et_al_2022_Figure 5/Fig 5/Fig 5C_images/nIs348;ctbp-1(n4784);otIs123/L4/Snap-373.tiff]

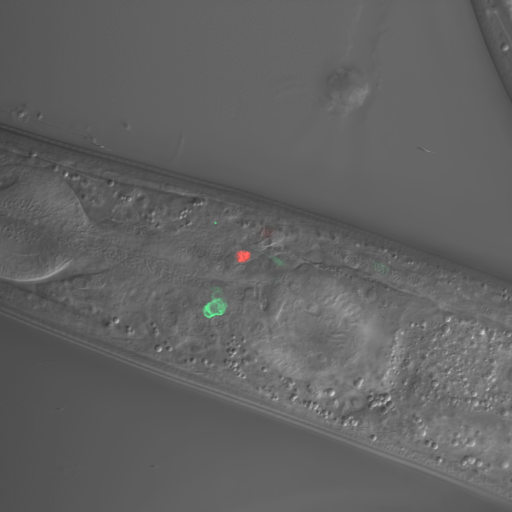

Supplement: Figure 5—source data 1. [file elife-74557-fig5-data1.zip › Saul_et_al_2022_Figure 5/Fig 5/Fig 5C_images/nIs348;ctbp-1(n4784);otIs123/L4/Snap-378.tiff]

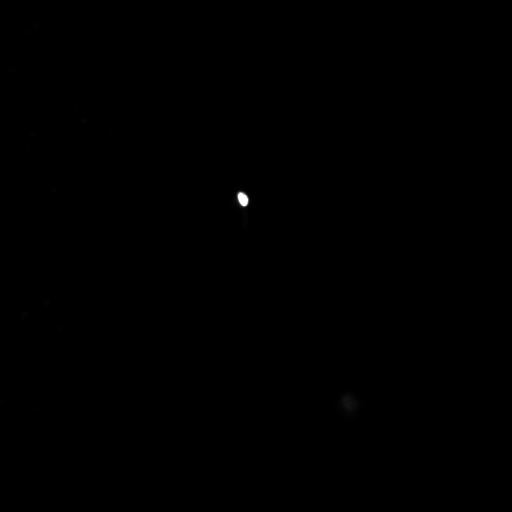

Supplement: Figure 5—source data 1. [file elife-74557-fig5-data1.zip › Saul_et_al_2022_Figure 5/Fig 5/Fig 5C_images/nIs348;ctbp-1(n4784);otIs123/L4/Snap-377.tiff_files/Snap-377_h0b0t0z0c2x0-512y0-512.tiff]

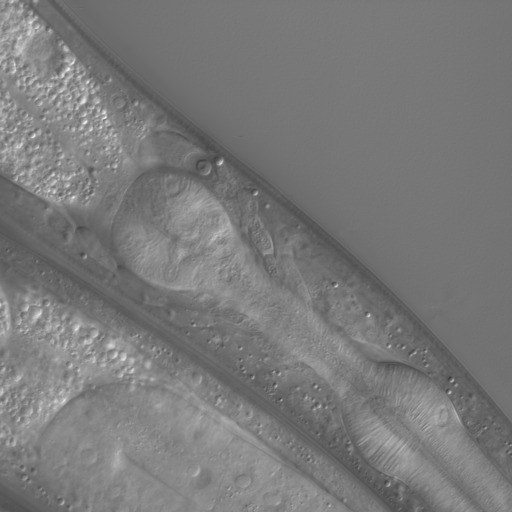

Supplement: Figure 5—source data 1. [file elife-74557-fig5-data1.zip › Saul_et_al_2022_Figure 5/Fig 5/Fig 5C_images/nIs348;ctbp-1(n4784);otIs123/L4/Snap-377.tiff_files/Snap-377_h0b0t0z0c1x0-512y0-512.tiff]

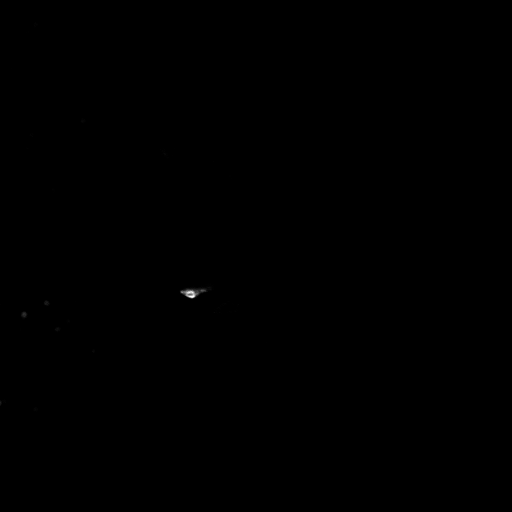

Supplement: Figure 5—source data 1. [file elife-74557-fig5-data1.zip › Saul_et_al_2022_Figure 5/Fig 5/Fig 5C_images/nIs348;ctbp-1(n4784);otIs123/L4/Snap-377.tiff_files/Snap-377_h0b0t0z0c0x0-512y0-512.tiff]

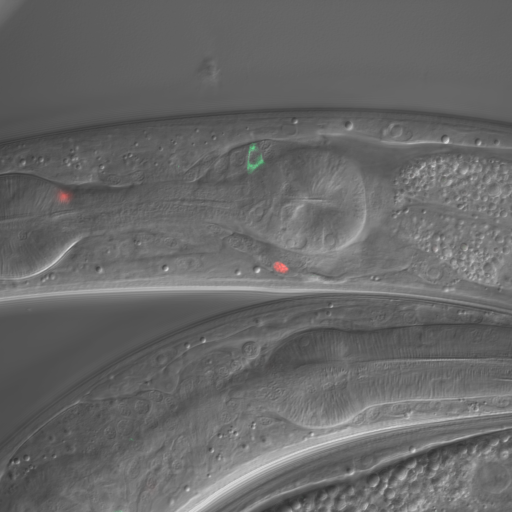

Supplement: Figure 5—source data 1. [file elife-74557-fig5-data1.zip › Saul_et_al_2022_Figure 5/Fig 5/Fig 5C_images/nIs348;ctbp-1(n4784);otIs123/L4/Snap-375.tiff]

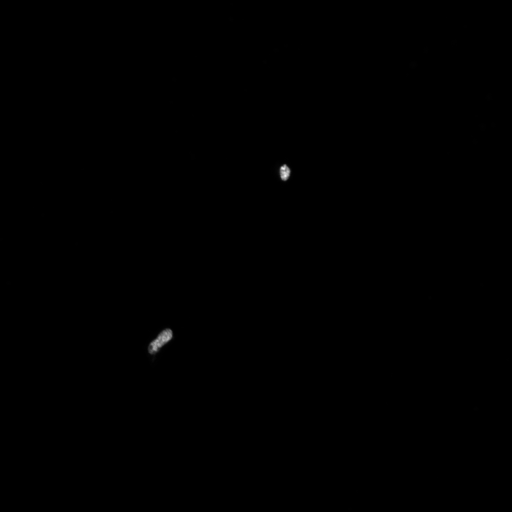

Supplement: Figure 5—source data 1. [file elife-74557-fig5-data1.zip › Saul_et_al_2022_Figure 5/Fig 5/Fig 5C_images/nIs348;ctbp-1(n4784);otIs123/L4/Snap-372.tiff_files/Snap-372_h0b0t0z0c2x0-512y0-512.tiff]

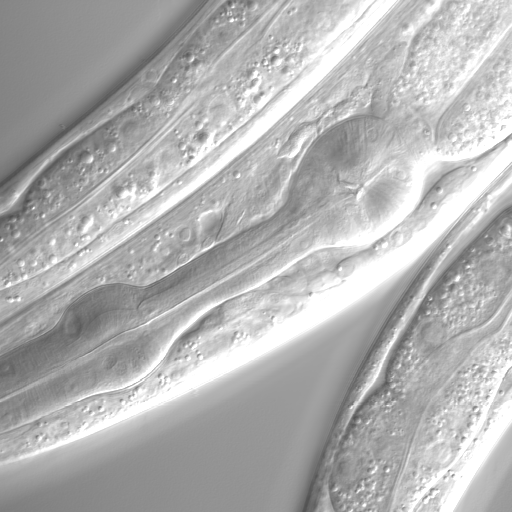

Supplement: Figure 5—source data 1. [file elife-74557-fig5-data1.zip › Saul_et_al_2022_Figure 5/Fig 5/Fig 5C_images/nIs348;ctbp-1(n4784);otIs123/L4/Snap-372.tiff_files/Snap-372_h0b0t0z0c1x0-512y0-512.tiff]

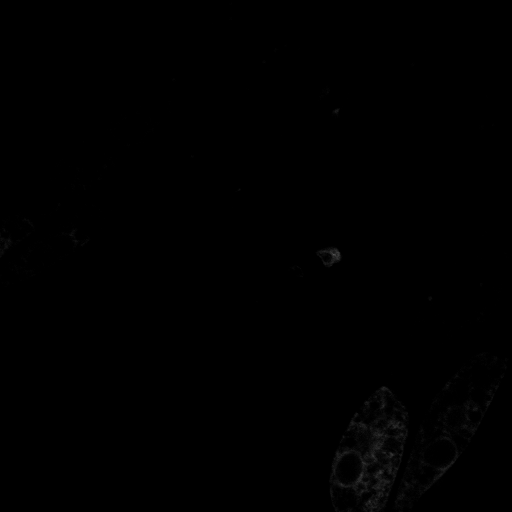

Supplement: Figure 5—source data 1. [file elife-74557-fig5-data1.zip › Saul_et_al_2022_Figure 5/Fig 5/Fig 5C_images/nIs348;ctbp-1(n4784);otIs123/L4/Snap-372.tiff_files/Snap-372_h0b0t0z0c0x0-512y0-512.tiff]

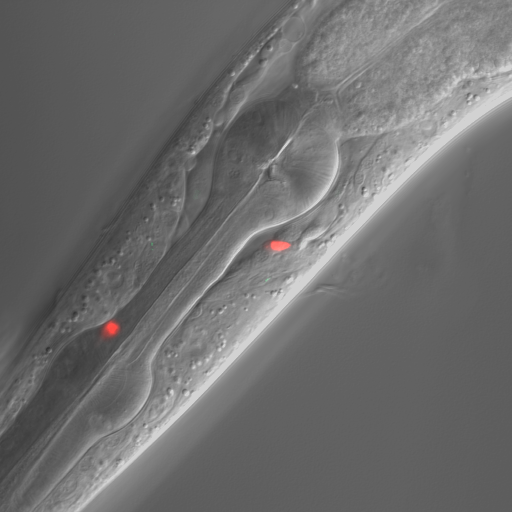

Supplement: Figure 5—source data 1. [file elife-74557-fig5-data1.zip › Saul_et_al_2022_Figure 5/Fig 5/Fig 5C_images/nIs348;ctbp-1(n4784);otIs123/L4/Snap-379.tiff]

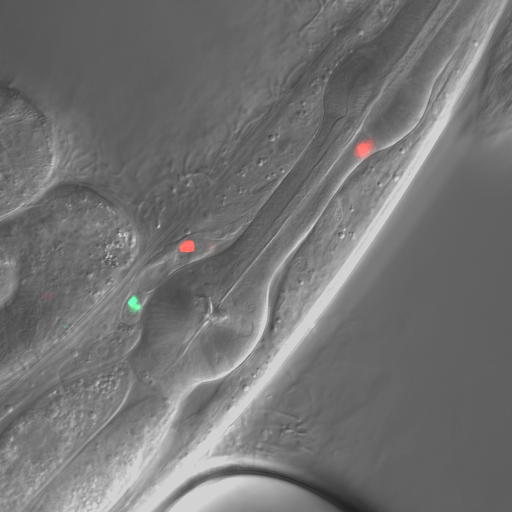

Supplement: Figure 5—source data 1. [file elife-74557-fig5-data1.zip › Saul_et_al_2022_Figure 5/Fig 5/Fig 5C_images/nIs348;ctbp-1(n4784);otIs123/L4/Snap-380.tiff]

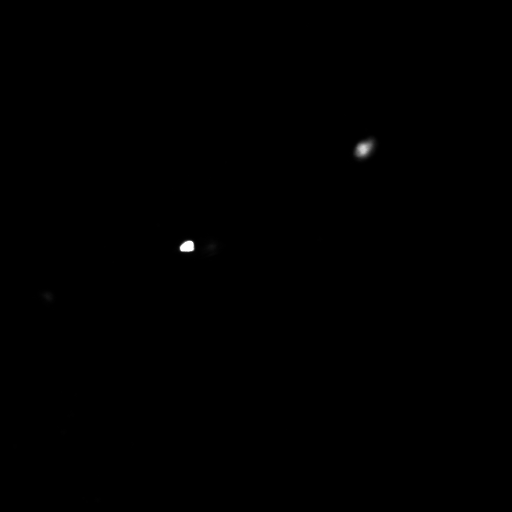

Supplement: Figure 5—source data 1. [file elife-74557-fig5-data1.zip › Saul_et_al_2022_Figure 5/Fig 5/Fig 5C_images/nIs348;ctbp-1(n4784);otIs123/L4/Snap-380.tiff_files/Snap-380_h0b0t0z0c2x0-512y0-512.tiff]

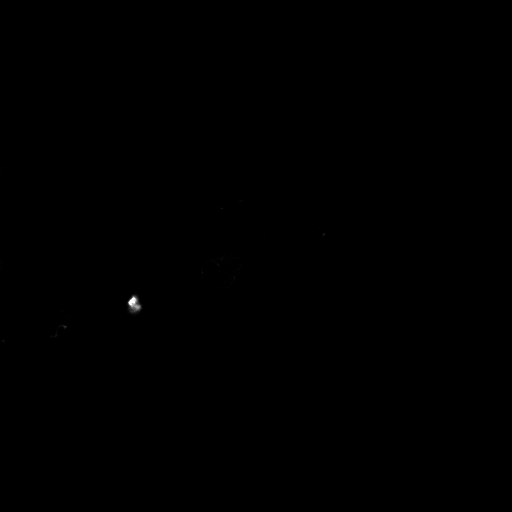

Supplement: Figure 5—source data 1. [file elife-74557-fig5-data1.zip › Saul_et_al_2022_Figure 5/Fig 5/Fig 5C_images/nIs348;ctbp-1(n4784);otIs123/L4/Snap-380.tiff_files/Snap-380_h0b0t0z0c0x0-512y0-512.tiff]

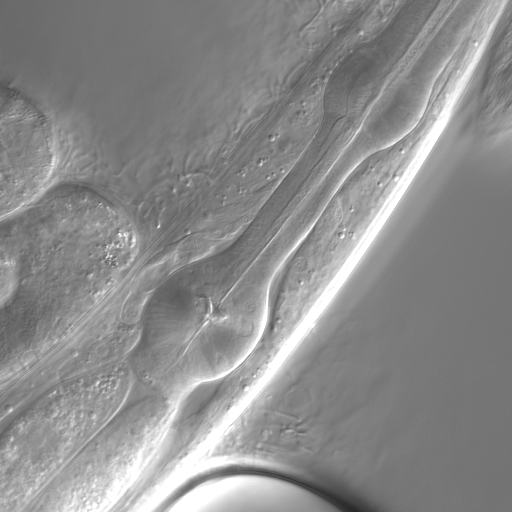

Supplement: Figure 5—source data 1. [file elife-74557-fig5-data1.zip › Saul_et_al_2022_Figure 5/Fig 5/Fig 5C_images/nIs348;ctbp-1(n4784);otIs123/L4/Snap-380.tiff_files/Snap-380_h0b0t0z0c1x0-512y0-512.tiff]

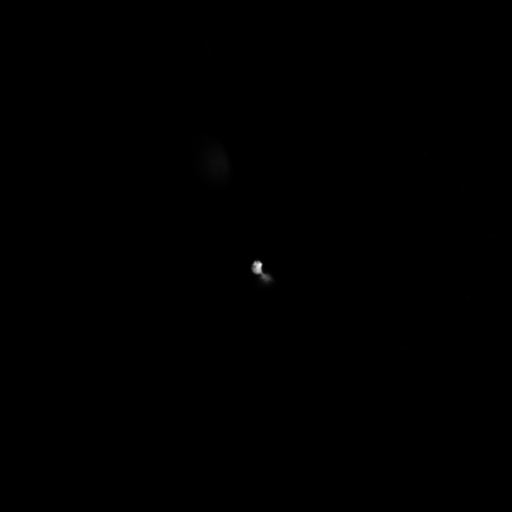

Supplement: Figure 5—source data 1. [file elife-74557-fig5-data1.zip › Saul_et_al_2022_Figure 5/Fig 5/Fig 5C_images/nIs348;ctbp-1(n4784);otIs123/L1/Snap-365.tiff_files/Snap-365_h0b0t0z0c2x0-512y0-512.tiff]

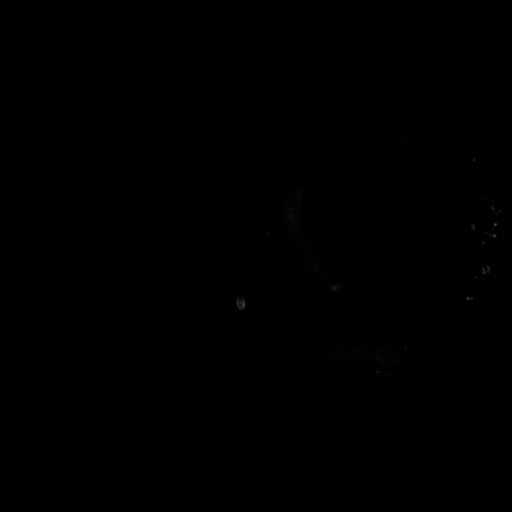

Supplement: Figure 5—source data 1. [file elife-74557-fig5-data1.zip › Saul_et_al_2022_Figure 5/Fig 5/Fig 5C_images/nIs348;ctbp-1(n4784);otIs123/L1/Snap-365.tiff_files/Snap-365_h0b0t0z0c0x0-512y0-512.tiff]

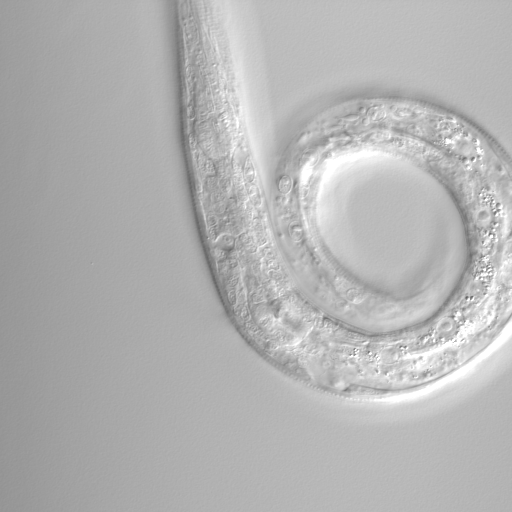

Supplement: Figure 5—source data 1. [file elife-74557-fig5-data1.zip › Saul_et_al_2022_Figure 5/Fig 5/Fig 5C_images/nIs348;ctbp-1(n4784);otIs123/L1/Snap-365.tiff_files/Snap-365_h0b0t0z0c1x0-512y0-512.tiff]

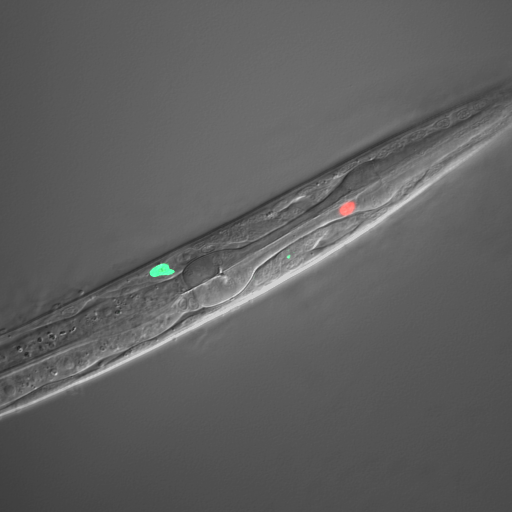

Supplement: Figure 5—source data 1. [file elife-74557-fig5-data1.zip › Saul_et_al_2022_Figure 5/Fig 5/Fig 5C_images/nIs348;ctbp-1(n4784);otIs123/L1/Snap-361.tiff]

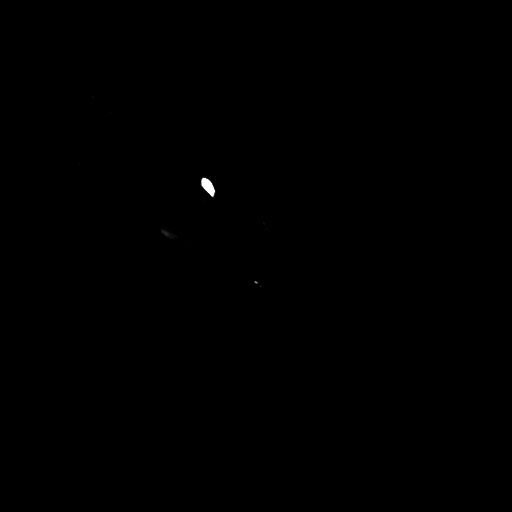

Supplement: Figure 5—source data 1. [file elife-74557-fig5-data1.zip › Saul_et_al_2022_Figure 5/Fig 5/Fig 5C_images/nIs348;ctbp-1(n4784);otIs123/L1/Snap-358.tiff_files/Snap-358_h0b0t0z0c0x0-512y0-512.tiff]

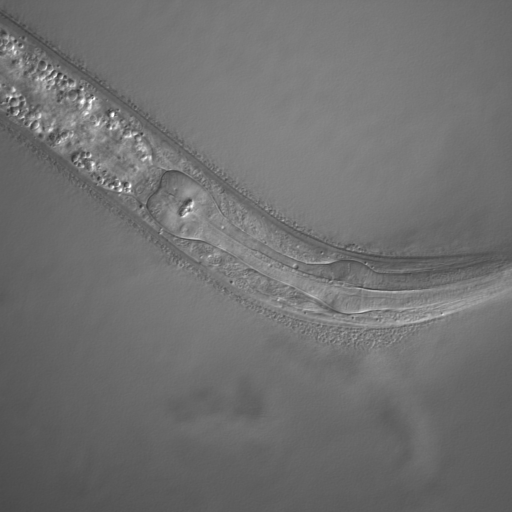

Supplement: Figure 5—source data 1. [file elife-74557-fig5-data1.zip › Saul_et_al_2022_Figure 5/Fig 5/Fig 5C_images/nIs348;ctbp-1(n4784);otIs123/L1/Snap-358.tiff_files/Snap-358_h0b0t0z0c1x0-512y0-512.tiff]

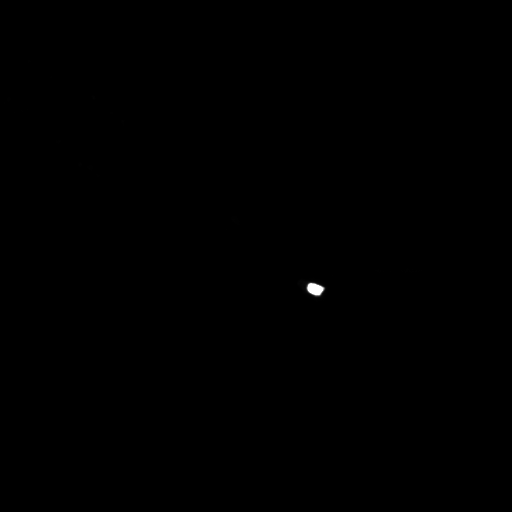

Supplement: Figure 5—source data 1. [file elife-74557-fig5-data1.zip › Saul_et_al_2022_Figure 5/Fig 5/Fig 5C_images/nIs348;ctbp-1(n4784);otIs123/L1/Snap-358.tiff_files/Snap-358_h0b0t0z0c2x0-512y0-512.tiff]

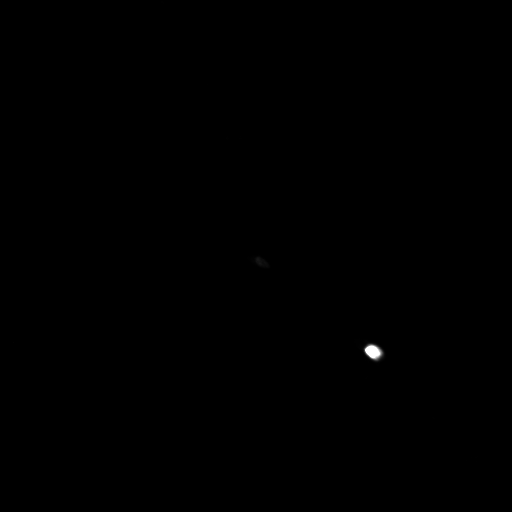

Supplement: Figure 5—source data 1. [file elife-74557-fig5-data1.zip › Saul_et_al_2022_Figure 5/Fig 5/Fig 5C_images/nIs348;ctbp-1(n4784);otIs123/L1/Snap-355.tiff_files/Snap-355_h0b0t0z0c2x0-512y0-512.tiff]

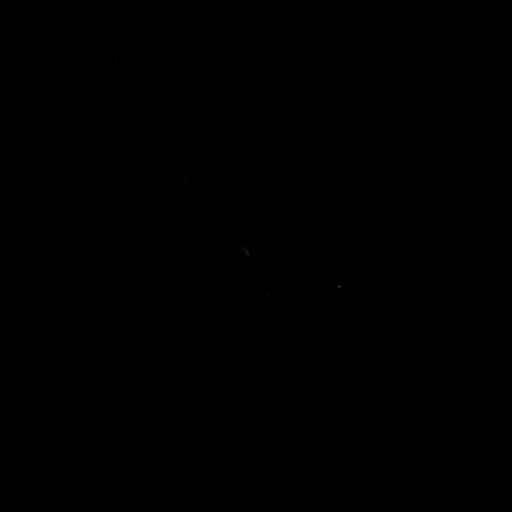

Supplement: Figure 5—source data 1. [file elife-74557-fig5-data1.zip › Saul_et_al_2022_Figure 5/Fig 5/Fig 5C_images/nIs348;ctbp-1(n4784);otIs123/L1/Snap-355.tiff_files/Snap-355_h0b0t0z0c0x0-512y0-512.tiff]

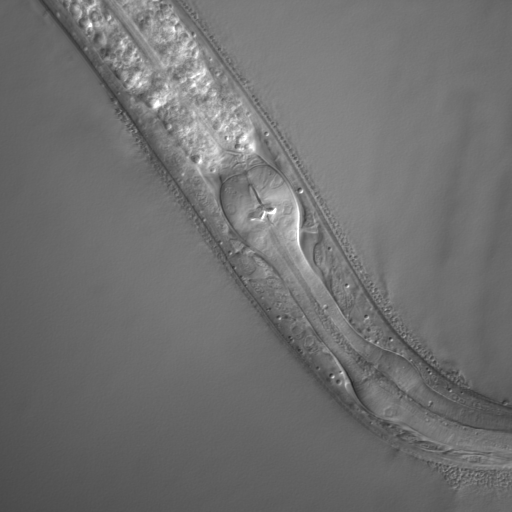

Supplement: Figure 5—source data 1. [file elife-74557-fig5-data1.zip › Saul_et_al_2022_Figure 5/Fig 5/Fig 5C_images/nIs348;ctbp-1(n4784);otIs123/L1/Snap-355.tiff_files/Snap-355_h0b0t0z0c1x0-512y0-512.tiff]

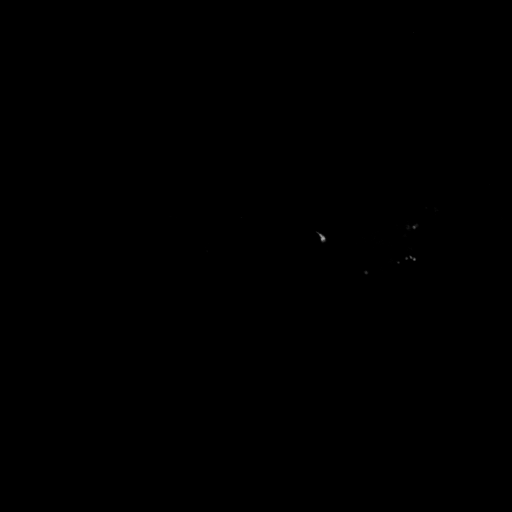

Supplement: Figure 5—source data 1. [file elife-74557-fig5-data1.zip › Saul_et_al_2022_Figure 5/Fig 5/Fig 5C_images/nIs348;ctbp-1(n4784);otIs123/L1/Snap-362.tiff_files/Snap-362_h0b0t0z0c0x0-512y0-512.tiff]

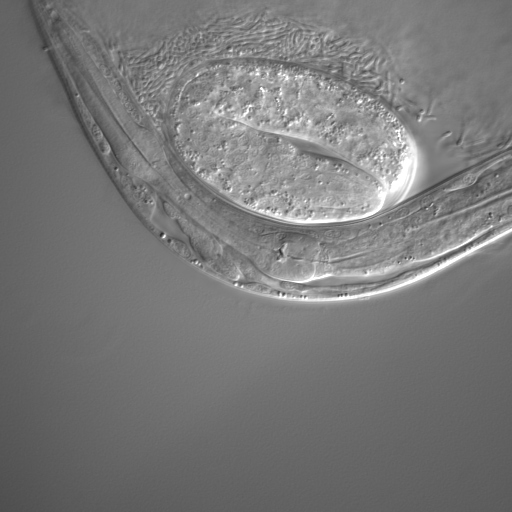

Supplement: Figure 5—source data 1. [file elife-74557-fig5-data1.zip › Saul_et_al_2022_Figure 5/Fig 5/Fig 5C_images/nIs348;ctbp-1(n4784);otIs123/L1/Snap-362.tiff_files/Snap-362_h0b0t0z0c1x0-512y0-512.tiff]

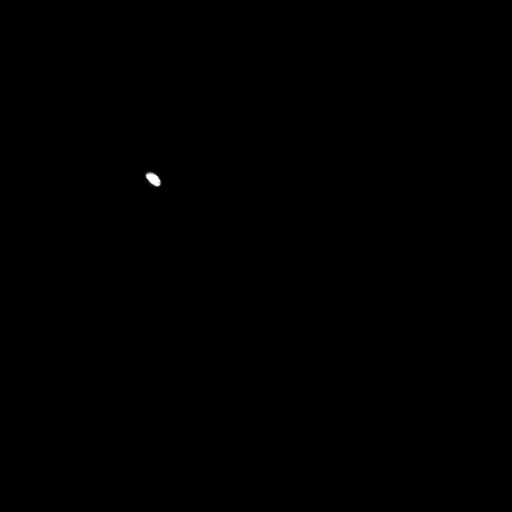

Supplement: Figure 5—source data 1. [file elife-74557-fig5-data1.zip › Saul_et_al_2022_Figure 5/Fig 5/Fig 5C_images/nIs348;ctbp-1(n4784);otIs123/L1/Snap-362.tiff_files/Snap-362_h0b0t0z0c2x0-512y0-512.tiff]

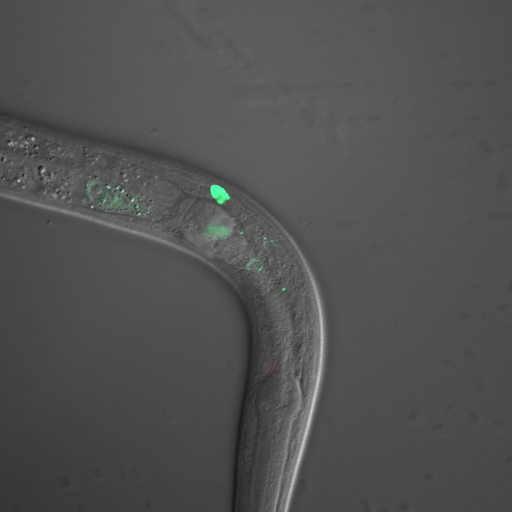

Supplement: Figure 5—source data 1. [file elife-74557-fig5-data1.zip › Saul_et_al_2022_Figure 5/Fig 5/Fig 5C_images/nIs348;ctbp-1(n4784);otIs123/L1/Snap-350.tiff]

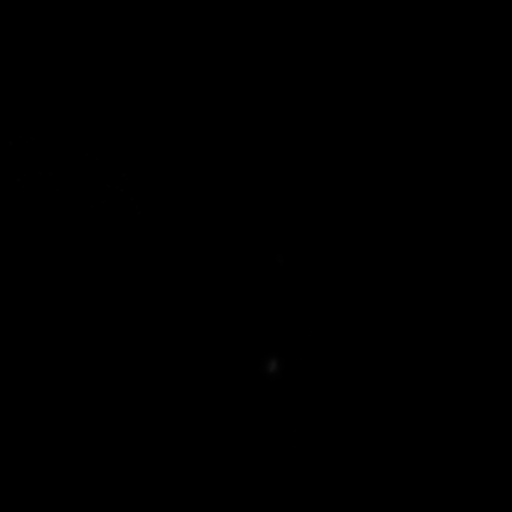

Supplement: Figure 5—source data 1. [file elife-74557-fig5-data1.zip › Saul_et_al_2022_Figure 5/Fig 5/Fig 5C_images/nIs348;ctbp-1(n4784);otIs123/L1/Snap-350.tiff_files/Snap-350_h0b0t0z0c2x0-512y0-512.tiff]

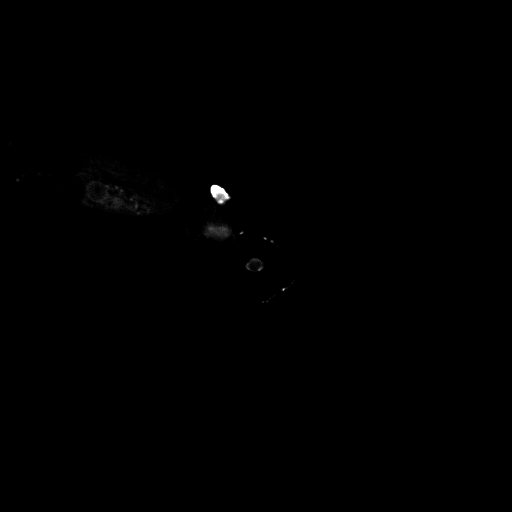

Supplement: Figure 5—source data 1. [file elife-74557-fig5-data1.zip › Saul_et_al_2022_Figure 5/Fig 5/Fig 5C_images/nIs348;ctbp-1(n4784);otIs123/L1/Snap-350.tiff_files/Snap-350_h0b0t0z0c0x0-512y0-512.tiff]

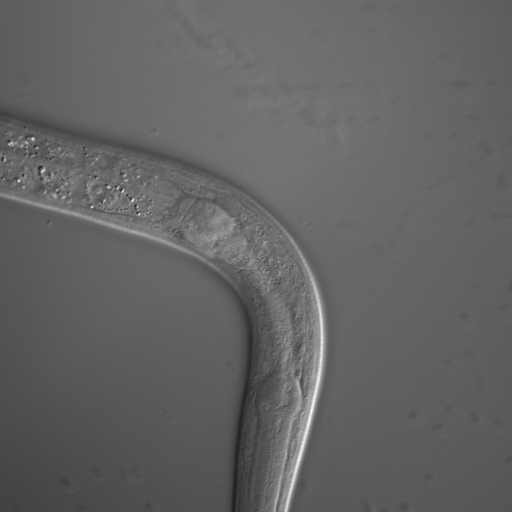

Supplement: Figure 5—source data 1. [file elife-74557-fig5-data1.zip › Saul_et_al_2022_Figure 5/Fig 5/Fig 5C_images/nIs348;ctbp-1(n4784);otIs123/L1/Snap-350.tiff_files/Snap-350_h0b0t0z0c1x0-512y0-512.tiff]

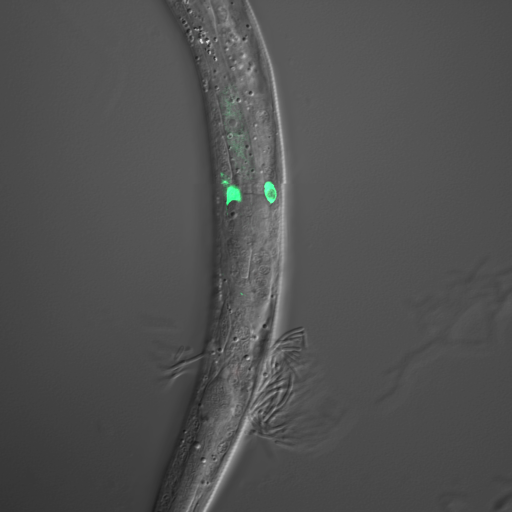

Supplement: Figure 5—source data 1. [file elife-74557-fig5-data1.zip › Saul_et_al_2022_Figure 5/Fig 5/Fig 5C_images/nIs348;ctbp-1(n4784);otIs123/L1/Snap-347.tiff]

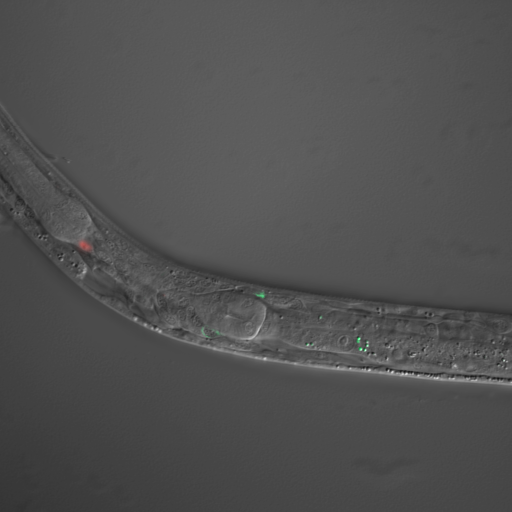

Supplement: Figure 5—source data 1. [file elife-74557-fig5-data1.zip › Saul_et_al_2022_Figure 5/Fig 5/Fig 5C_images/nIs348;ctbp-1(n4784);otIs123/L1/Snap-348.tiff]

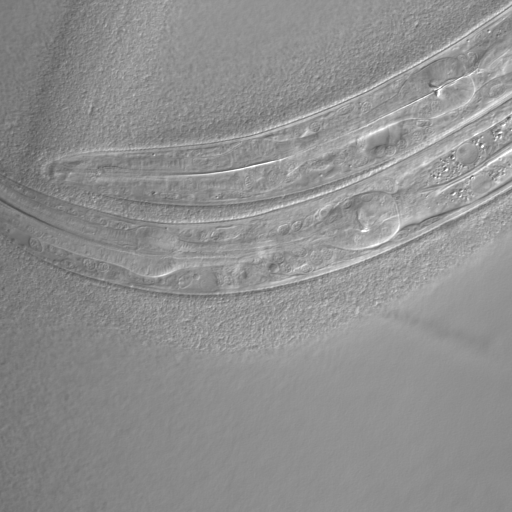

Supplement: Figure 5—source data 1. [file elife-74557-fig5-data1.zip › Saul_et_al_2022_Figure 5/Fig 5/Fig 5C_images/nIs348;ctbp-1(n4784);otIs123/L1/Snap-369.tiff_files/Snap-369_h0b0t0z0c1x0-512y0-512.tiff]

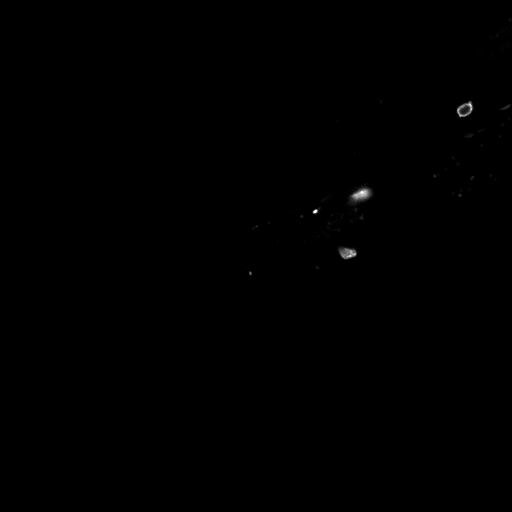

Supplement: Figure 5—source data 1. [file elife-74557-fig5-data1.zip › Saul_et_al_2022_Figure 5/Fig 5/Fig 5C_images/nIs348;ctbp-1(n4784);otIs123/L1/Snap-369.tiff_files/Snap-369_h0b0t0z0c0x0-512y0-512.tiff]

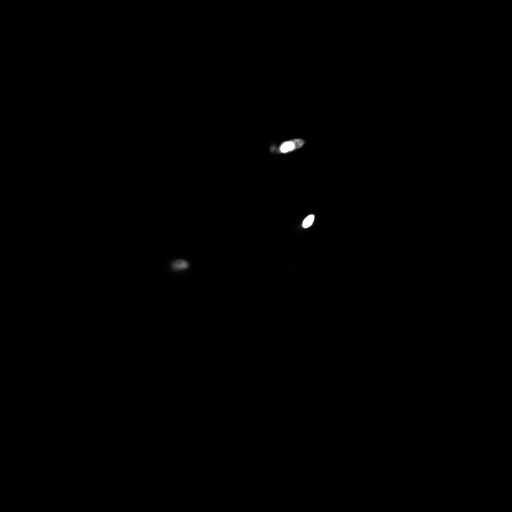

Supplement: Figure 5—source data 1. [file elife-74557-fig5-data1.zip › Saul_et_al_2022_Figure 5/Fig 5/Fig 5C_images/nIs348;ctbp-1(n4784);otIs123/L1/Snap-369.tiff_files/Snap-369_h0b0t0z0c2x0-512y0-512.tiff]

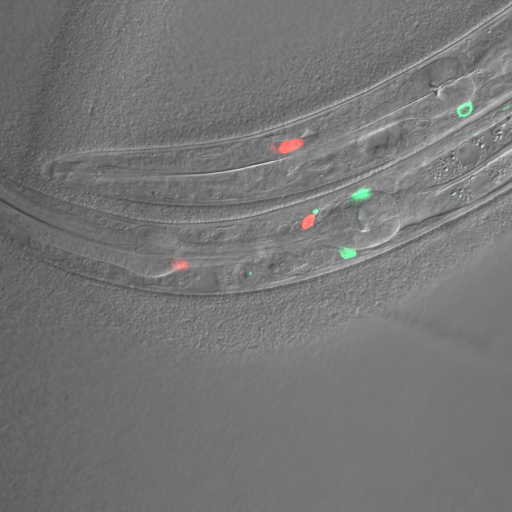

Supplement: Figure 5—source data 1. [file elife-74557-fig5-data1.zip › Saul_et_al_2022_Figure 5/Fig 5/Fig 5C_images/nIs348;ctbp-1(n4784);otIs123/L1/Snap-369.tiff]

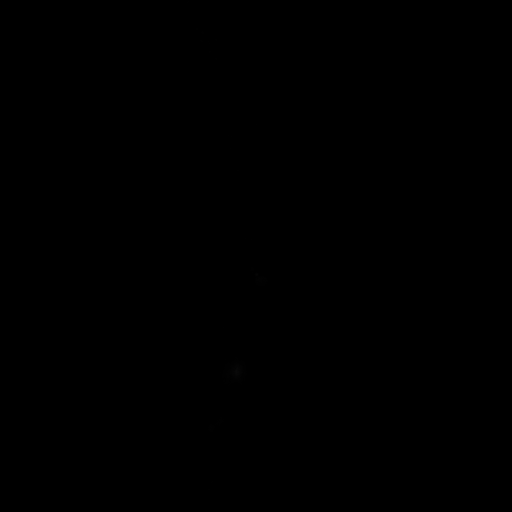

Supplement: Figure 5—source data 1. [file elife-74557-fig5-data1.zip › Saul_et_al_2022_Figure 5/Fig 5/Fig 5C_images/nIs348;ctbp-1(n4784);otIs123/L1/Snap-347.tiff_files/Snap-347_h0b0t0z0c2x0-512y0-512.tiff]

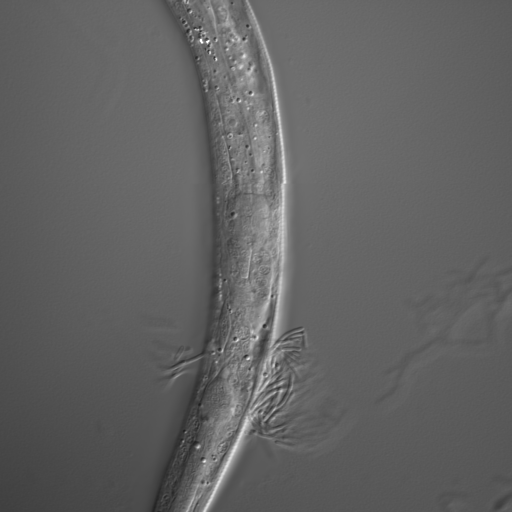

Supplement: Figure 5—source data 1. [file elife-74557-fig5-data1.zip › Saul_et_al_2022_Figure 5/Fig 5/Fig 5C_images/nIs348;ctbp-1(n4784);otIs123/L1/Snap-347.tiff_files/Snap-347_h0b0t0z0c1x0-512y0-512.tiff]

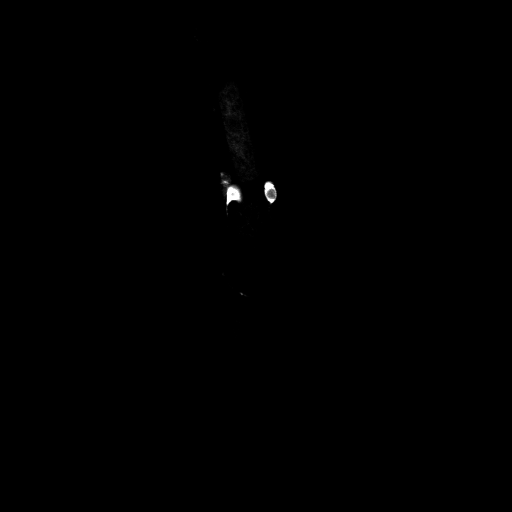

Supplement: Figure 5—source data 1. [file elife-74557-fig5-data1.zip › Saul_et_al_2022_Figure 5/Fig 5/Fig 5C_images/nIs348;ctbp-1(n4784);otIs123/L1/Snap-347.tiff_files/Snap-347_h0b0t0z0c0x0-512y0-512.tiff]

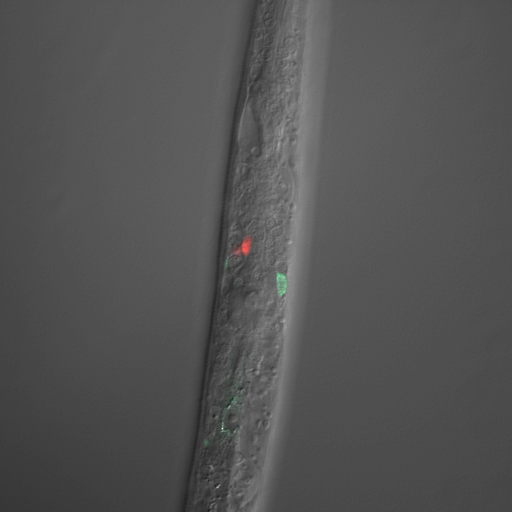

Supplement: Figure 5—source data 1. [file elife-74557-fig5-data1.zip › Saul_et_al_2022_Figure 5/Fig 5/Fig 5C_images/nIs348;ctbp-1(n4784);otIs123/L1/Snap-345.tiff]

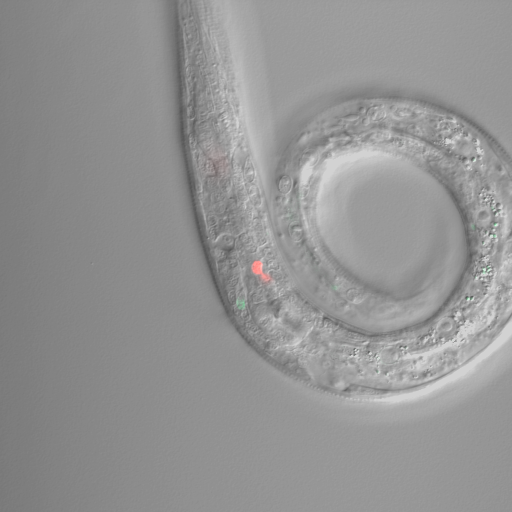

Supplement: Figure 5—source data 1. [file elife-74557-fig5-data1.zip › Saul_et_al_2022_Figure 5/Fig 5/Fig 5C_images/nIs348;ctbp-1(n4784);otIs123/L1/Snap-365.tiff]

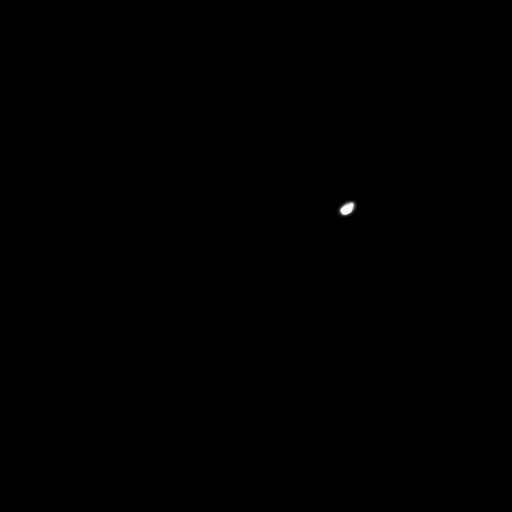

Supplement: Figure 5—source data 1. [file elife-74557-fig5-data1.zip › Saul_et_al_2022_Figure 5/Fig 5/Fig 5C_images/nIs348;ctbp-1(n4784);otIs123/L1/Snap-361.tiff_files/Snap-361_h0b0t0z0c2x0-512y0-512.tiff]

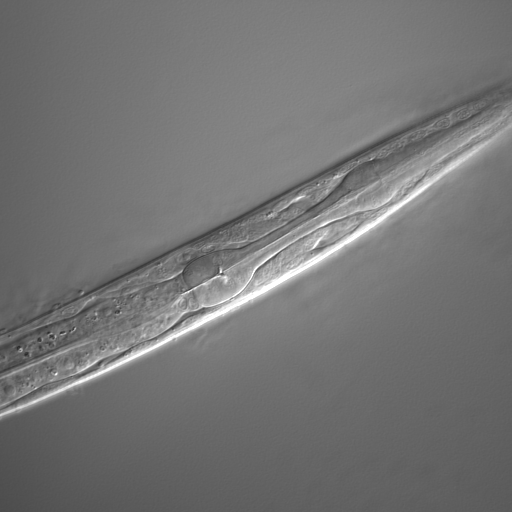

Supplement: Figure 5—source data 1. [file elife-74557-fig5-data1.zip › Saul_et_al_2022_Figure 5/Fig 5/Fig 5C_images/nIs348;ctbp-1(n4784);otIs123/L1/Snap-361.tiff_files/Snap-361_h0b0t0z0c1x0-512y0-512.tiff]

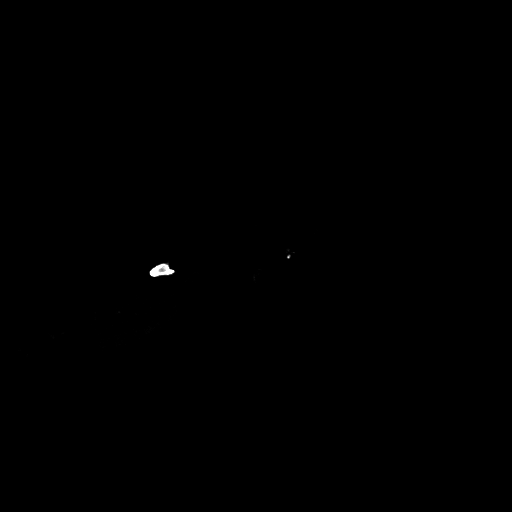

Supplement: Figure 5—source data 1. [file elife-74557-fig5-data1.zip › Saul_et_al_2022_Figure 5/Fig 5/Fig 5C_images/nIs348;ctbp-1(n4784);otIs123/L1/Snap-361.tiff_files/Snap-361_h0b0t0z0c0x0-512y0-512.tiff]

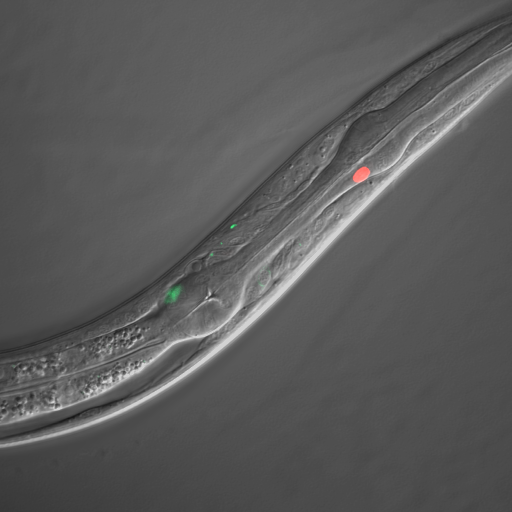

Supplement: Figure 5—source data 1. [file elife-74557-fig5-data1.zip › Saul_et_al_2022_Figure 5/Fig 5/Fig 5C_images/nIs348;ctbp-1(n4784);otIs123/L1/Snap-354.tiff]

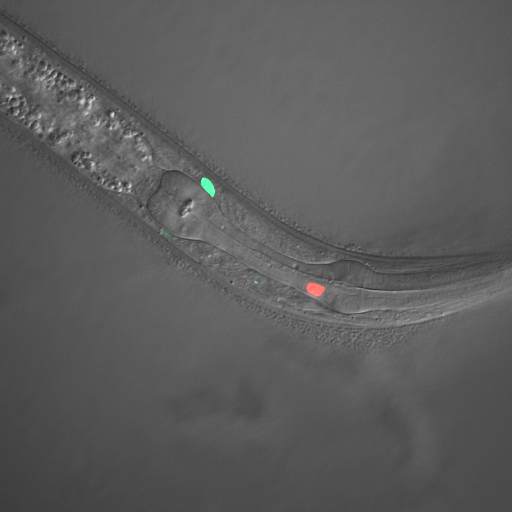

Supplement: Figure 5—source data 1. [file elife-74557-fig5-data1.zip › Saul_et_al_2022_Figure 5/Fig 5/Fig 5C_images/nIs348;ctbp-1(n4784);otIs123/L1/Snap-358.tiff]

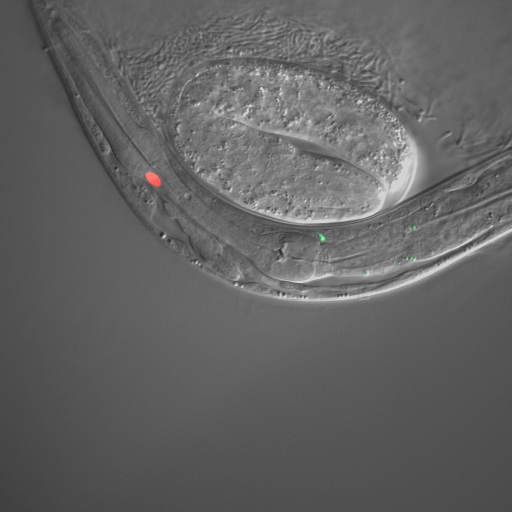

Supplement: Figure 5—source data 1. [file elife-74557-fig5-data1.zip › Saul_et_al_2022_Figure 5/Fig 5/Fig 5C_images/nIs348;ctbp-1(n4784);otIs123/L1/Snap-362.tiff]

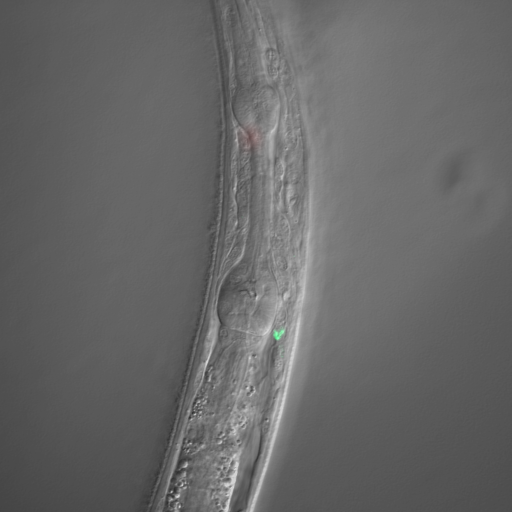

Supplement: Figure 5—source data 1. [file elife-74557-fig5-data1.zip › Saul_et_al_2022_Figure 5/Fig 5/Fig 5C_images/nIs348;ctbp-1(n4784);otIs123/L1/Snap-363.tiff]

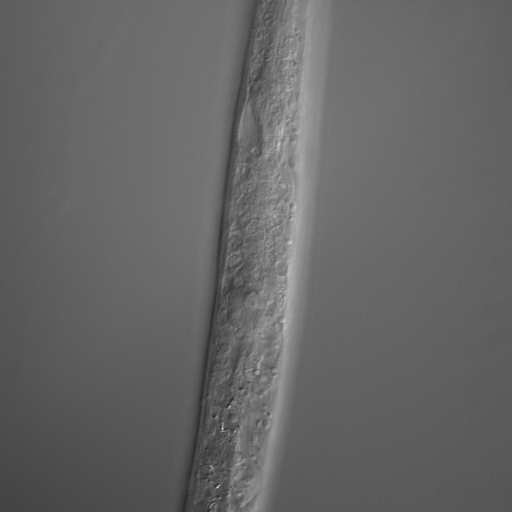

Supplement: Figure 5—source data 1. [file elife-74557-fig5-data1.zip › Saul_et_al_2022_Figure 5/Fig 5/Fig 5C_images/nIs348;ctbp-1(n4784);otIs123/L1/Snap-345.tiff_files/Snap-345_h0b0t0z0c1x0-512y0-512.tiff]

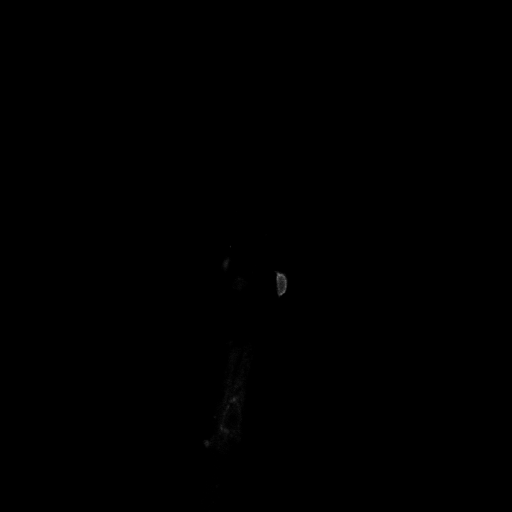

Supplement: Figure 5—source data 1. [file elife-74557-fig5-data1.zip › Saul_et_al_2022_Figure 5/Fig 5/Fig 5C_images/nIs348;ctbp-1(n4784);otIs123/L1/Snap-345.tiff_files/Snap-345_h0b0t0z0c0x0-512y0-512.tiff]

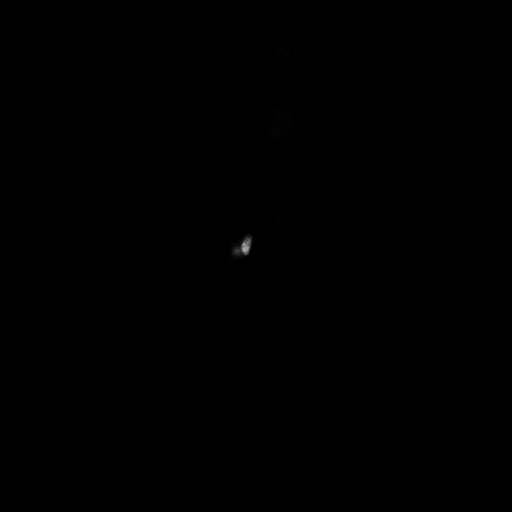

Supplement: Figure 5—source data 1. [file elife-74557-fig5-data1.zip › Saul_et_al_2022_Figure 5/Fig 5/Fig 5C_images/nIs348;ctbp-1(n4784);otIs123/L1/Snap-345.tiff_files/Snap-345_h0b0t0z0c2x0-512y0-512.tiff]

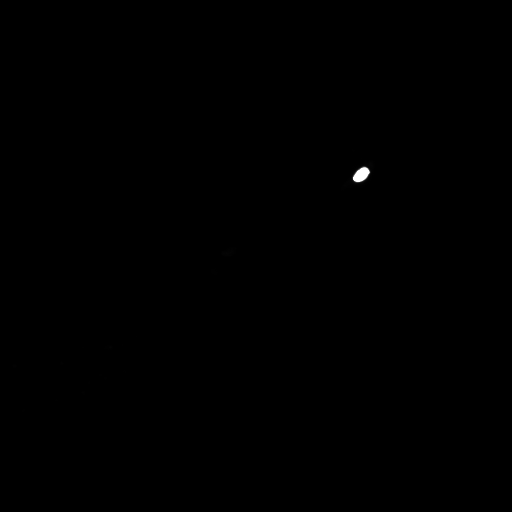

Supplement: Figure 5—source data 1. [file elife-74557-fig5-data1.zip › Saul_et_al_2022_Figure 5/Fig 5/Fig 5C_images/nIs348;ctbp-1(n4784);otIs123/L1/Snap-354.tiff_files/Snap-354_h0b0t0z0c2x0-512y0-512.tiff]

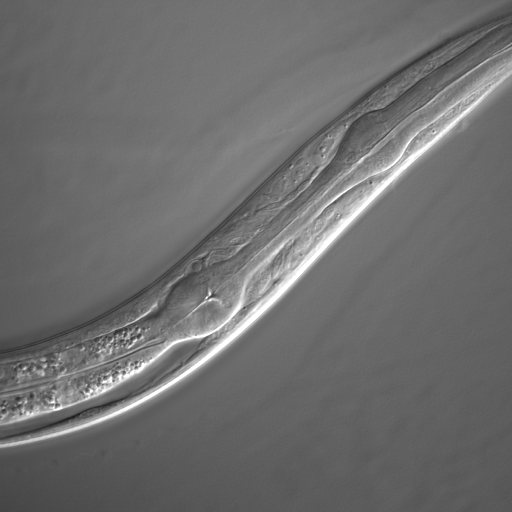

Supplement: Figure 5—source data 1. [file elife-74557-fig5-data1.zip › Saul_et_al_2022_Figure 5/Fig 5/Fig 5C_images/nIs348;ctbp-1(n4784);otIs123/L1/Snap-354.tiff_files/Snap-354_h0b0t0z0c1x0-512y0-512.tiff]

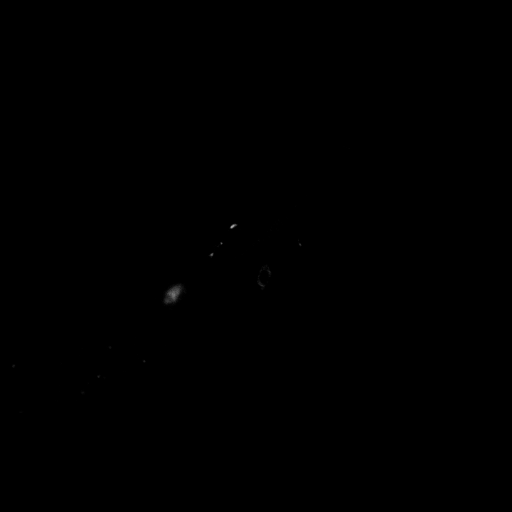

Supplement: Figure 5—source data 1. [file elife-74557-fig5-data1.zip › Saul_et_al_2022_Figure 5/Fig 5/Fig 5C_images/nIs348;ctbp-1(n4784);otIs123/L1/Snap-354.tiff_files/Snap-354_h0b0t0z0c0x0-512y0-512.tiff]

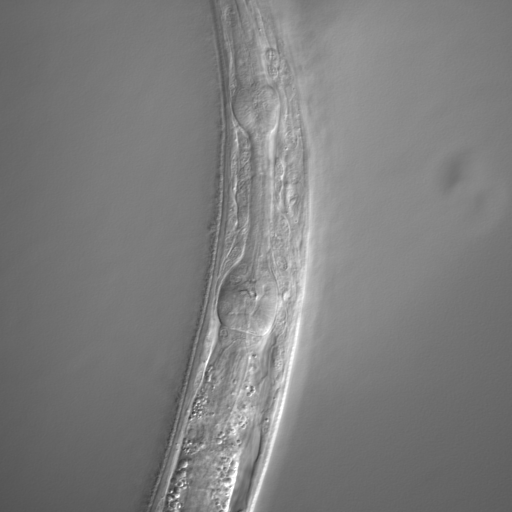

Supplement: Figure 5—source data 1. [file elife-74557-fig5-data1.zip › Saul_et_al_2022_Figure 5/Fig 5/Fig 5C_images/nIs348;ctbp-1(n4784);otIs123/L1/Snap-363.tiff_files/Snap-363_h0b0t0z0c1x0-512y0-512.tiff]

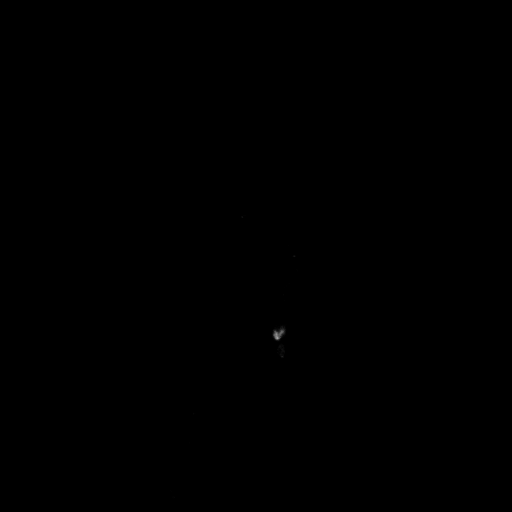

Supplement: Figure 5—source data 1. [file elife-74557-fig5-data1.zip › Saul_et_al_2022_Figure 5/Fig 5/Fig 5C_images/nIs348;ctbp-1(n4784);otIs123/L1/Snap-363.tiff_files/Snap-363_h0b0t0z0c0x0-512y0-512.tiff]

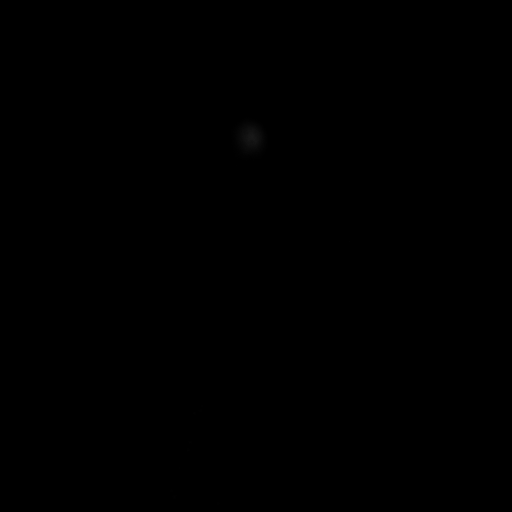

Supplement: Figure 5—source data 1. [file elife-74557-fig5-data1.zip › Saul_et_al_2022_Figure 5/Fig 5/Fig 5C_images/nIs348;ctbp-1(n4784);otIs123/L1/Snap-363.tiff_files/Snap-363_h0b0t0z0c2x0-512y0-512.tiff]

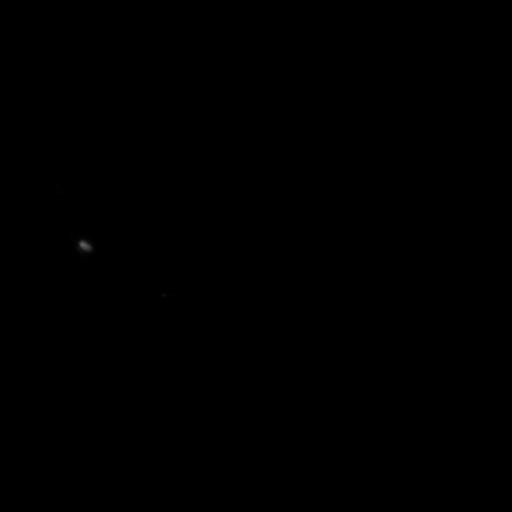

Supplement: Figure 5—source data 1. [file elife-74557-fig5-data1.zip › Saul_et_al_2022_Figure 5/Fig 5/Fig 5C_images/nIs348;ctbp-1(n4784);otIs123/L1/Snap-348.tiff_files/Snap-348_h0b0t0z0c2x0-512y0-512.tiff]

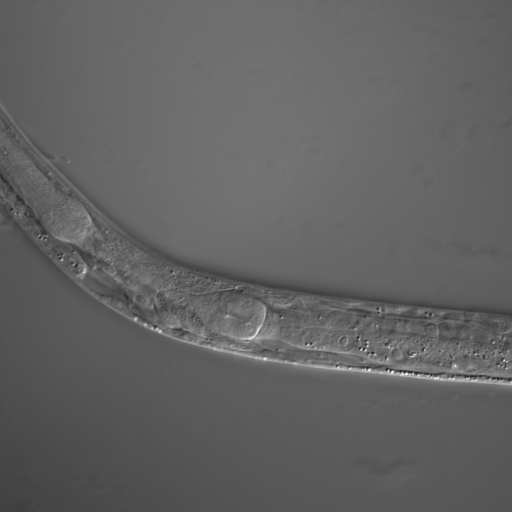

Supplement: Figure 5—source data 1. [file elife-74557-fig5-data1.zip › Saul_et_al_2022_Figure 5/Fig 5/Fig 5C_images/nIs348;ctbp-1(n4784);otIs123/L1/Snap-348.tiff_files/Snap-348_h0b0t0z0c1x0-512y0-512.tiff]

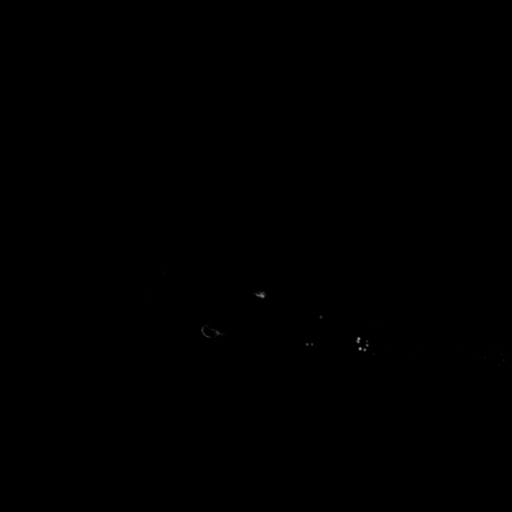

Supplement: Figure 5—source data 1. [file elife-74557-fig5-data1.zip › Saul_et_al_2022_Figure 5/Fig 5/Fig 5C_images/nIs348;ctbp-1(n4784);otIs123/L1/Snap-348.tiff_files/Snap-348_h0b0t0z0c0x0-512y0-512.tiff]

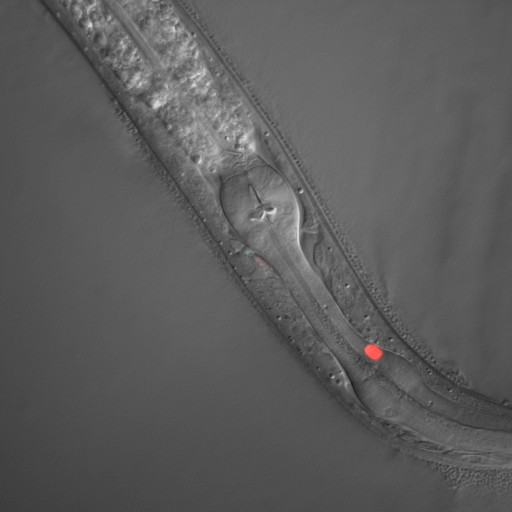

Supplement: Figure 5—source data 1. [file elife-74557-fig5-data1.zip › Saul_et_al_2022_Figure 5/Fig 5/Fig 5C_images/nIs348;ctbp-1(n4784);otIs123/L1/Snap-355.tiff]

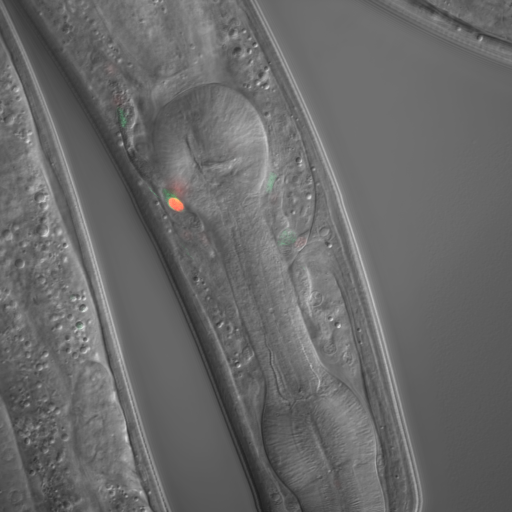

Supplement: Figure 5—source data 1. [file elife-74557-fig5-data1.zip › Saul_et_al_2022_Figure 5/Fig 5/Fig 5C_images/nIs843;otIs123/L4/Snap-340.tiff]

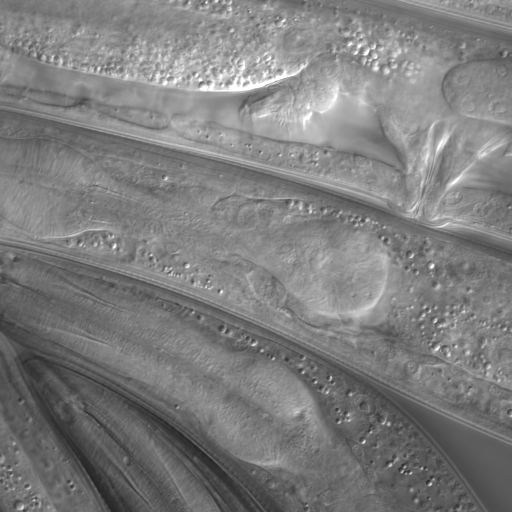

Supplement: Figure 5—source data 1. [file elife-74557-fig5-data1.zip › Saul_et_al_2022_Figure 5/Fig 5/Fig 5C_images/nIs843;otIs123/L4/Snap-338.tiff_files/Snap-338_h0b0t0z0c1x0-512y0-512.tiff]

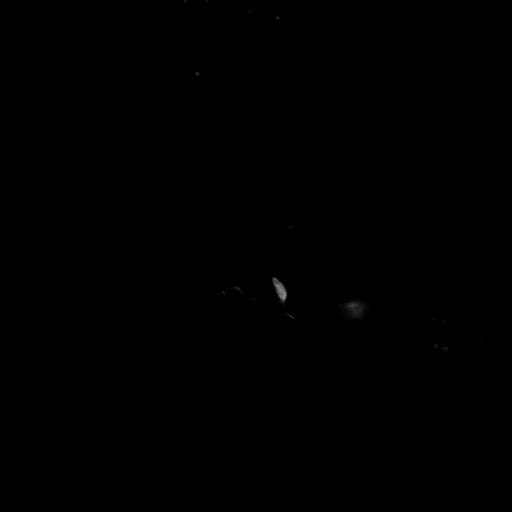

Supplement: Figure 5—source data 1. [file elife-74557-fig5-data1.zip › Saul_et_al_2022_Figure 5/Fig 5/Fig 5C_images/nIs843;otIs123/L4/Snap-338.tiff_files/Snap-338_h0b0t0z0c0x0-512y0-512.tiff]

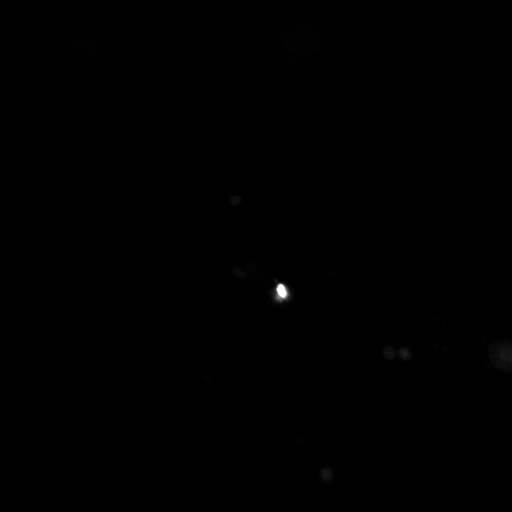

Supplement: Figure 5—source data 1. [file elife-74557-fig5-data1.zip › Saul_et_al_2022_Figure 5/Fig 5/Fig 5C_images/nIs843;otIs123/L4/Snap-338.tiff_files/Snap-338_h0b0t0z0c2x0-512y0-512.tiff]

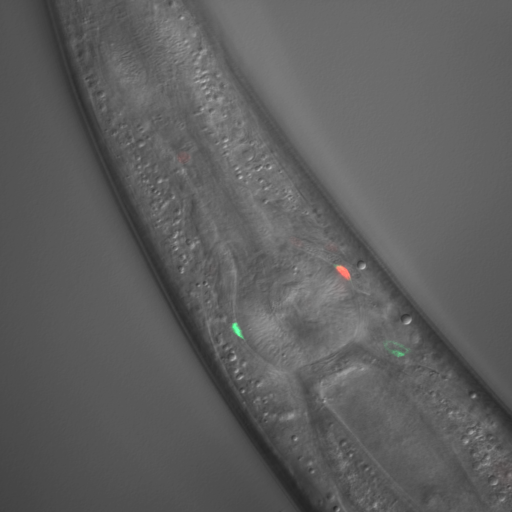

Supplement: Figure 5—source data 1. [file elife-74557-fig5-data1.zip › Saul_et_al_2022_Figure 5/Fig 5/Fig 5C_images/nIs843;otIs123/L4/Snap-337.tiff]

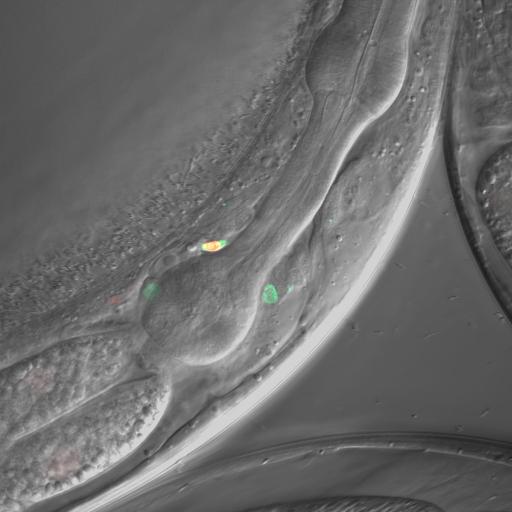

Supplement: Figure 5—source data 1. [file elife-74557-fig5-data1.zip › Saul_et_al_2022_Figure 5/Fig 5/Fig 5C_images/nIs843;otIs123/L4/Snap-336.tiff]

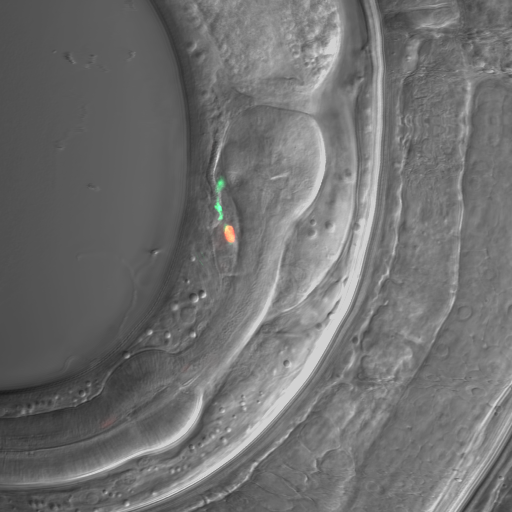

Supplement: Figure 5—source data 1. [file elife-74557-fig5-data1.zip › Saul_et_al_2022_Figure 5/Fig 5/Fig 5C_images/nIs843;otIs123/L4/Snap-331.tiff]

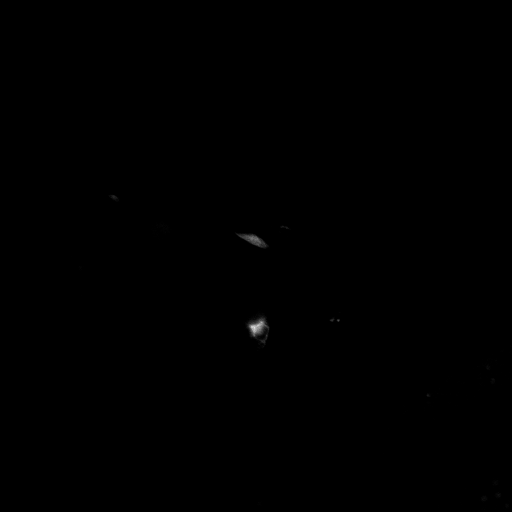

Supplement: Figure 5—source data 1. [file elife-74557-fig5-data1.zip › Saul_et_al_2022_Figure 5/Fig 5/Fig 5C_images/nIs843;otIs123/L4/Snap-344.tiff_files/Snap-344_h0b0t0z0c0x0-512y0-512.tiff]

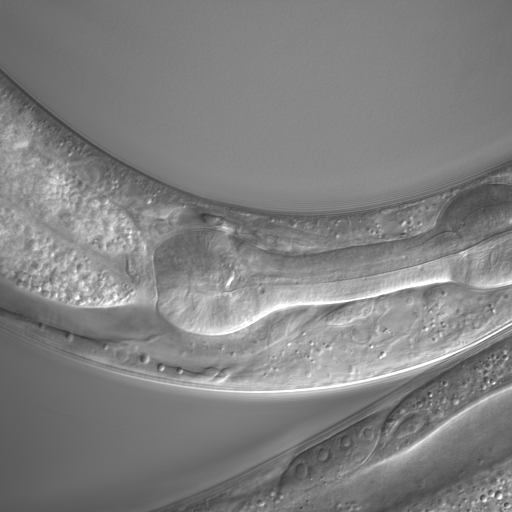

Supplement: Figure 5—source data 1. [file elife-74557-fig5-data1.zip › Saul_et_al_2022_Figure 5/Fig 5/Fig 5C_images/nIs843;otIs123/L4/Snap-344.tiff_files/Snap-344_h0b0t0z0c1x0-512y0-512.tiff]

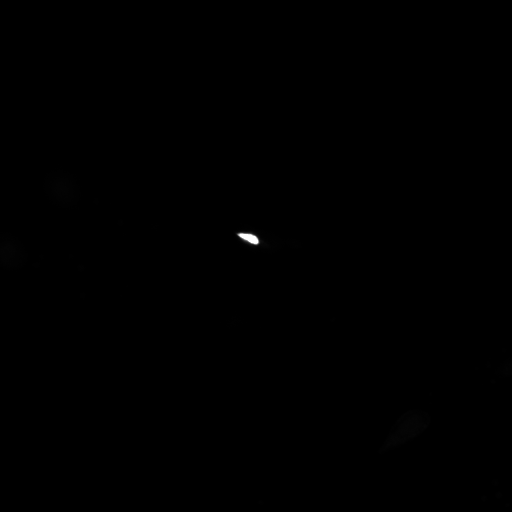

Supplement: Figure 5—source data 1. [file elife-74557-fig5-data1.zip › Saul_et_al_2022_Figure 5/Fig 5/Fig 5C_images/nIs843;otIs123/L4/Snap-344.tiff_files/Snap-344_h0b0t0z0c2x0-512y0-512.tiff]

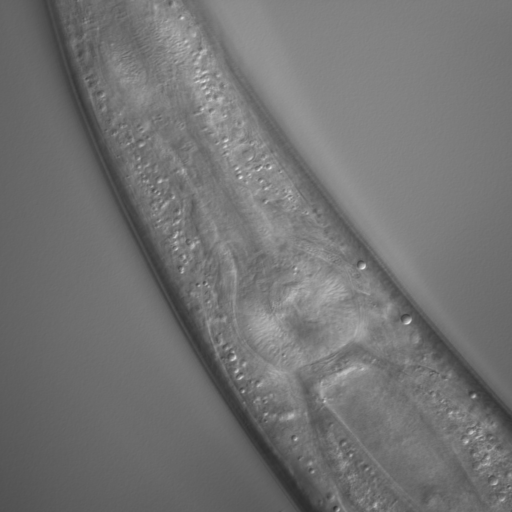

Supplement: Figure 5—source data 1. [file elife-74557-fig5-data1.zip › Saul_et_al_2022_Figure 5/Fig 5/Fig 5C_images/nIs843;otIs123/L4/Snap-337.tiff_files/Snap-337_h0b0t0z0c1x0-512y0-512.tiff]
